# Simultaneous extraction and quantitative analysis of *S*-Methyl-l-Cysteine Sulfoxide, sulforaphane and glucosinolates in cruciferous vegetables by liquid chromatography mass spectrometry

**DOI:** 10.1016/j.fochx.2023.101065

**Published:** 2023-12-14

**Authors:** Armaghan Shafaei, Caroline R. Hill, Jonathan M. Hodgson, Lauren C. Blekkenhorst, Mary C. Boyce

**Affiliations:** a Centre for Integrative Metabolomics & Computational Biology, School of Science, Edith Cowan University, Joondalup, Western Australia, Australia; b Nutrition and Health Innovation Research Institute, School of Medical and Health Sciences, Edith Cowan University, Perth, Western Australia, Australia; c Royal Perth Hospital Research Foundation, Perth, Australia; d School of Science, Edith Cowan University, Joondalup, Western Australia, Australia

**Keywords:** Cruciferous, Brassica, Glucosinolates, Sulforaphane, S-methyl-l-cysteine sulfoxide, LC-MS, 4-methoxyglucobrassicin (PubChem CID: 656563), Glucobrassicanapin (PubChem CID: 5485207), Glucobrassicin (PubChem CID: 5484743), Glucoiberin (PubChem CID: 954862), Glucoerucin (PubChem CID: 656539), Gluconapin (PubChem CID: 9548620), Gluconasturtiin (PubChem CID: 656555), Glucoraphanin (PubChem CID: 9548634), Progoitrin (PubChem CID: 5281139), Sinigrin (PubChem CID: 23682211), S-methyl cysteine sulfoxide: (PubChem CID: 82142).

## Abstract

Sulfur containing compounds including glucosinolates (GLS), sulforaphane (SFN) and S-methyl-l-cysteine sulfoxide (SMCSO) have been proposed to be partly responsible for the beneficial health effects of cruciferous vegetables. As such, greater understanding of their measurements within foods is important to estimate intake in humans and to inform dietary intervention studies. Herein is described a simple and sensitive method for simultaneous analysis of 20 GLS, SFN and SMCSO by liquid chromatography mass spectrometry. Analytes were effectively retained and resolved on an Xbridge C18 column. Detection can be achieved using high resolution or unit resolution mass spectrometry; the latter making the method more applicable to large studies. Quantitative analysis using calibration standards was demonstrated for 10 GLS, SFN and SMCSO. A further 10 GLS were tentatively identified using high resolution mass spectrometry. The use of surrogate GLS standards was shown to be unreliable, with closely related GLS displaying significantly different ionisation efficiencies.

## Highlights

• Liquid chromatography-mass spectrometry method for quantification of sulfur-containing compounds in cruciferous vegetables.
• Simultaneous determination of glucosinolates, sulforaphane and S-methyl-l-cysteine sulfoxide in cruciferous vegetables.
• Comparison of performance of a high resolution and unit resolution mass spectrometers.
• Glucosinolates, sulforaphane and S-methyl-l-cysteine sulfoxide quantifications in raw and cooked cruciferous vegetables.

## 1 Introduction

Cruciferous vegetables belong to the genus *Brassica*. They are promoted as part of a healthy diet as they are a rich source of nutritive and non-nutritive bioactive compounds, that have antioxidant, anti-inflammatory and anti-cancer properties (Almushayti et al., 2021, Armah et al., 2015, Blekkenhorst et al., 2018, Mocniak et al., 2023, Orlando et al., 2022). Many of these beneficial health properties are attributed to the relative abundance of sulfur containing compounds, such as glucosinolates (GLS) and their metabolites, most notably sulforaphane (SFN), and cysteine sulfoxides, such as *S*-methyl-l-cysteine sulfoxide (SMCSO) (Hill et al., 2022).

Glucosinolates are secondary plant metabolites that consist of a β-d-thioglucoside attached to a sulfonated moiety and a variable amino acid-derived side chain (Almushayti et al., 2021). Depending on the structure of this amino acid-derived side chain, GLS are classed as aliphatic, aromatic or indole. Over 130 different GLS are found in plants and are often found at higher levels in cruciferous vegetables (Blažević et al., 2020, Mocniak et al., 2023, Orlando et al., 2022). On cutting, crushing or chewing of the cruciferous vegetables, the thioglucoside linkage in the GLS is hydrolysed by the enzyme myrosinase leading to the formation of glucose and an unstable thiohydroximate-*O*-sulfonate (Almushayti et al., 2021). The resulting unstable thiohydroximate-*O*-sulfonate quickly converts to one of several biologically active isothiocyanates (Künstler, Gullner, Ádám, Kolozsváriné Nagy, & Király, 2020). In addition to isothiocyanates, other hydrolysis products, such as thiocyanates, nitriles, and epithionitriles, can be formed through the hydrolysis of GLS. However, the formation of these compounds typically requires the presence of specifier proteins along with myrosinase (Blažević et al., 2020).

SFN is an aliphatic isothiocyanate derived from the hydrolysis of the GSL glucoraphanin, during the process described in the previous paragraph (Bello, Maldini, Baima, Scaccini, & Natella, 2018). As the release of SFN occurs when vegetables are damaged or cut, whole, intact and healthy plants generally contain minimal SFN content. Furthermore, low SFN concentrations are indicative of fresh, high-quality, undamaged vegetables with maximum retention of intact GLS (Westphal et al., 2017). In nutrition studies aimed at quantifying and comparing GLS concentrations in different vegetables, monitoring SFN becomes crucial to ensure minimal loss of GLS through their conversion to SFN. Therefore, SFN could serve as a useful marker for freshness of the selected vegetables (Campas-Baypoli, Sánchez-Machado, Bueno-Solano, Ramírez-Wong, & López-Cervantes, 2010).

SMCSO (or methiin) is an *S*-alk(en)yl cysteine sulfoxide found in relatively high abundance within *Brassica* vegetables (1–4 % dry weight), and interestingly at even greater levels than GLS (0.1–0.6 % dry weight) (Hill et al., 2022). SMCSO has demonstrated several protective and inhibitory effects such as antioxidant against hypercholesterolemic-induced damage, anti-hyperglycemic, anti-inflammatory and VLDL-cholesterol lowering properties, anti-obesogenic and anti-microbial effects principally *in vitro* and in animal models (Bagiu et al., 2012, Castro et al., 2021, Itokawa et al., 1973, Kumari and Augusti, 2002, Kumari and Augusti, 2007, Lemos et al., 2021, Tanaka et al., 2014, Yoshinari et al., 2012). Additionally, the steroidogenic potential of SMCSO has also been explored (Nakayama, Ho, Yamagishi, Ikemoto, Komai, & Shirakawa, 2020).

Emerging evidence of the health benefits of GLS and SMCSO has increased the demands to monitor and measure concentrations of these bioactive compounds within foods. For example, Wu, Chen, Yu, Chen, Ye, and Zhang (2021) monitored the impact of different cooking methods upon GLS concentrations within red cabbage; Li et al. (2021) analysed the varying GLS profiles of 80 different broccoli genotypes; whilst Yu, Ma, Zhang, and Li (2020) explored the presence of 18 intact GLS in 15 *Brassicaceae* vegetables. More recently, Friedrich, Wermter, Andernach, Witzel, and Hanschen (2022) monitored the SMCSO concentrations in commercially available cabbages over a three-month period. Recognising the health potential of GLS and SMCSO, a greater understanding of their measurements within foods is important to estimate intake in humans and to inform dietary intervention studies. Furthermore, to achieve precise GLS measurement, it is crucial to incorporate SFN into the analysis, enabling verification of the freshness and integrity of the raw vegetable samples obtained.

Liquid chromatography – mass spectrometry (LC-MS) has become the method of choice for the analysis of GLS. It avoids the tedious and poorly controlled desulfation step evident in earlier studies (Hooshmand and Fomsgaard, 2021, Hwang et al., 2019; Kim et al., 2022, Liang et al., 2018, Wu et al., 2021). The separation of GLS is usually accomplished using a C18 column, with mobile phases consisting of either methanol or acetonitrile and acidified water, and their detection achieved using triple quadrupole mass spectrometry (QQQ-MS) (Hooshmand et al., 2021; Kim et al., 2022, Liang et al., 2018) or high-resolution mass spectrometry (HR-MS) (Hooshmand et al., 2021; Hwang et al., 2019, Li et al., 2021, Shi et al., 2017). LC-MS has also been implemented for quantification of GLS metabolites such as SFN in cruciferous vegetables; Bello et al. (2018) reported the separation and detection of SFN in broccoli juices using a C18 column and MS detection.

Several papers report LC-MS as the technique of choice for analysing other sulfur-based compounds including SMCSO in vegetables and biological samples (Joshi, Renaud, Sumarah, & Marsolais, 2019; Kim et al., 2016, Matsutomo and Kodera, 2016, Rektorisova et al., 2020, Sivapalan et al., 2019). Separation of SMCSO can be achieved using acidified water and acetonitrile/methanol on a C18, (Kim et al., 2016) or Amide column (Rektorisova et al., 2020). The use of an ion pairing reagent in combination with a C18 column has also been reported for better sensitivity and peak shape of SMCSO and related compounds in human biological samples (Sivapalan et al., 2019).

Despite the large number of available analytical procedures for the analysis of GLS, SMCSO and SFN, there is no reporting of a simple, sensitive, and reliable method for simultaneous identification and quantification of these important sulfur-based compounds. We hypothesized that it was possible to develop a single method for the separation and quantitative analysis of GLS, SFN and SMCSO extracted from cruciferous vegetables using LC-MS. To test this hypothesis, we aimed to develop (i) a chromatographic separation method that effectively retained the analytes, and resolved known GLS isomers and isobars, SFN and SMCSO whilst validating the separation using a second separation column that employed an alternative mechanism of separation and (ii) a quantitative detection method using mass spectrometry. To the best of our knowledge, we report here for the first time a method for the simultaneous determination of 20 GLS, SMCSO and SFN using HR-MS and showcasing its suitability for analysing various raw and cooked cruciferous vegetables. Additionally, we expand the method’s applicability by incorporating QQQ-MS detection, thereby enhancing its feasibility for large studies involving many samples.

## 2 Materials and methods

### 2.1 Chemicals and reagents

LC-MS grade water, formic acid, acetic acid, acetonitrile and methanol were purchased from Thermo Fisher Scientific (Sydney, Australia). Analytical reagent grade ammonium formate and ammonium acetate were purchased from Chem Supply (Adelaide, Australia). *S*-methyl-l-cysteine sulfoxide (SMCSO), deuterated *S*-methyl-l-cysteine sulfoxide (*d3*-SMCSO), d,l-sulforaphane (SFN), deuterated d,l-sulforaphane (*d8*-SFN) were obtained from Toronto Research Chemicals (Toronto, Canada). Gluconapin (GNA), glucoiberin (GIB), glucoerucin (GER), progoitrin (PRO), glucobrassicanapin (GBN), glucobrassicin (GBR), glucoraphanin (GRA), gluconasturtiin (GNS), sinigrin (SIN), and glucotropaeolin (GTR) were purchased from PhytoLab GmbH & Co. (Vestenbergsgreuth, Germany) and 4-methoxyglucobrassicin (MGB) was obtained from Medical Isotopes (NH, USA).

### 2.2 Preparation of standard mixture and calibration standards

Individual stock solutions (1 mg/mL) of 10 GLS (GNA, GIB, GER, PRO, GBN, GBR, GRA, GNS, SIN and MGB), SMCSO and SFN were prepared in water or methanol and stored at −80 °C until use. Mix calibration standard solutions (ten) in the range 0.02–5 µg/mL for GNA, GIB, GER, PRO, GBN, GBR, GRA, GNS, SIN and MGB, 0.008–2 µg/mL for SFN and 0.4–100 µg/mL for SMCSO were prepared in 0.1 % aqueous formic acid (C18 method) and acetonitrile (HILIC method).

The internal standards (GTR, *d3*-SMCSO and *d8*-SFN) were prepared in methanol at a concentration of 1 mg/mL and stored in a −80 °C freezer. An internal standard solution (100 µg/mL) containing GTR, *d3*-SMCSO and *d8*-SFN were prepared in LC-MS grade water.

### 2.3 Analytical conditions

#### 2.3.1 Optimisation of stationary phase

Multiple columns from various manufacturers including ACQUITY UPLC BEH Amide column (100 × 2.1 mm packed with 1.7 µm particles, Waters), a Syncronis™ HILIC column (100 × 2.1 mm packed with 1.7 µm particles, Thermo Scientific), an ACE C18 PFP column (100 × 2.1 mm packed with 1.7 µm particles, Advanced Chromatography Technologies, Scotland), an ACE C18 column (100 × 2.1 mm packed with 1.7 µm particles, Advanced Chromatography Technologies, Scotland) and an XBridge C18 column (100 × 3.0 mm packed with 3.5 µm particles, Waters) were chosen for testing the separation of GLS, SMCSO and SFN. The reversed phase stationary phases (C18 PFP, C18 and XBridge C18) were tested using 2 different mobile phases: (1) water and methanol both containing 0.1 % formic acid, and (2) water and acetonitrile both containing 0.1 % formic acid. For HILIC stationary phases (BEH Amide and Syncronis™ or Zic HILIC), two mobile phases at different pH were used: (1) water and acetonitrile both containing 10 mM ammonium formate adjusted to pH 3 with formic acid, and (2) water and acetonitrile both containing 10 mM ammonium acetate adjusted to pH 5 with acetic acid.

#### 2.3.2 LC HR-MS analysis

Chromatographic separation was performed on a Thermo Scientific Ultimate 3000 Liquid Chromatography coupled to a Thermo Scientific Q Exactive Focus Orbitrap mass spectrometer equipped with an ESI source. Analysis of vegetable extracts on LC HR-MS was achieved on a Xbridge C18 column, using a mobile phase of water containing 0.1 % formic acid (A) and acetonitrile containing 0.1 % formic acid (B). The initial mobile phase conditions were 99.9 % A and 0.1 % B. The linear gradient was as follows: 0–1 min, 0.1 % B; 1–6 min, 0.1–50 % B; 6–9 min, 50–99.9 %; 9–12 min, 99.9 % B; 12–12.5 min, 99.9–0.1 % B; 12.5–15 min 0.1 % B. The flow rate was 0.5 mL/minute, and the column temperature was maintained at 35 °C. The sample injection volume was 4 µL and the auto sampler was maintained at 6 °C.

Full-scan in combination with MS^2^ analysis was performed using a Q Exactive Focus mass spectrometer. Electrospray ionisation in negative and positive ion modes was used with a spray voltage of 2500 V in negative mode and 3500 V in positive mode, an auxiliary gas flow rate of 14, sheath gas flow rate of 53, sweep gas flow rate of 3, capillary temperature of 269 °C, S-lense RF level of 50 and heater temperature of 438 °C. All quantitative data were acquired using the following settings: resolution = 70,000; automatic gain control (AGC) target = 1 × 10^6^; maximum injection time = auto; scan range = 50–600 *m*/*z*. The resolution of MS^2^ mode was set to 17,500 FWHM with AGC target set at 5 × 10^4^ and an isolation window of 1.0 *m*/*z*. The *m*/*z* of precursor ion for each analyte was used for identification and quantification in full-scan mode. The retention times and MS/MS patterns of the chemical standards in the MS^2^ mode were used for confirmation (Table 1). In addition to the 10 GLS compounds with available chemical standards, an additional 10 GLS compounds were identified without corresponding chemical standards. The process of tentative identification of these compounds involved comparing the *m*/*z* values of precursor ions and associated MS/MS patterns of each GLS identified in the samples with reported values for GLS in relevant literature (Table S1).

**Table 1:** Optimised LC-MS/MS parameters for 21 glucosinolates, sulforaphane and *S*-methyl-l-cysteine sulfoxide. The **base ions** are highlighted in bold.

Compound name	Chemical formula	Molecular weight (g/mol)	Adduct	LC HR-MS			LC-QQQ		
				Precursor ion (*m*/*z*)	Fragments	SNCE^1^	Precursor ion (*m*/*z*)	Fragments	CE^2^
**Aliphatic GLS**									
Glucoiberin (GIB)	C_11_H_21_NO_10_S_3_	423.480	[M−H]^-^	422.0255	74.99, 79.95, 95.95, **96.96,** 195.03, 259.01, 358.03	10, 20, 30	422.03	**97**	28
								196	25
								229	23
								259	22
Glucoraphanin (GRA)	C_12_H_23_NO_10_S_3_	437.493	[M−H]^-^	436.0411	74.99, 95.95, **96.96,** 178.02, 195.03, 259.01, 274.99, 372.04	10, 20, 30	436.16	97	28
								178	25
								259	22
								**372**	18
Glucoerucin (GER)	C_12_H_23_NO_9_S_3_	421.494	[M−H]^-^	420.0462	74.99, 79.96, 95.95, **96.96,** 174.04, 195.03, 259.01, 420.04, 274.99	10, 20, 30	420.06	**97**	28
								227	25
								242	23
								259	22
Glucocheirolin (GOC)	C_11_H_21_NO_11_S_3_	439.465	[M−H]^-^	438.0203	74.99, **96.96,** 135.97, 195.03, 259.01, 332.01	10, 20, 30	438.02	**97**	28
								259	22
Glucoberteroin (GOB)	C_13_H_24_NO_9_S_3_	435.538	[M−H]^-^	434.0619	74.99, **96.96,** 119.04, 128.93, 195.03, 214.00, 259.01, 274.99	10, 20, 30	434.06	**97**	28
								129	25
								195	25
								259	22
								354	19
Progoitrin (PRO)	C_11_H_19_NO_10_S_2_	389.390	[M−H]^-^	388.0378	74.99, 95.95, **96.96,** 135.97, 195.03, 259.01	10, 20, 30	388.26	**97**	28
								136	22
								259	22
								275	22
								331	14
Sinigrin (SIN)	C_10_H_17_NO_9_S_2_	359.373	[M−H]^-^	358.0272	74.99, **96.96,** 116.02, 161.99, 195.03, 259.01	10, 20, 30	358.14	**97**	28
								119	24
								129	22
								195	22
								259	22
								275	17
Gluconapin (GNA)	C_11_H_19_NO_9_S_2_	373.391	[M−H]^-^	372.0429	74.99, **96.96,** 119.04, 130.03, 145.05, 178.98, 195.03, 259.01, 274.99	10, 20, 30	371.94	**97**	28
								259	22
								325	19
Glucobrassicanapin (GBN)	C_12_H_21_NO_9_S_2_	387.418	[M−H]^-^	386.0585	74.99, **96.96,** 119.04, 128.93, 144.05, 195.03, 208.03, 227.02, 259.01, 274.99, 350.36	10, 20, 30	386.21	**97**	28
								193	22
								195	22
								259	22
Glucoraphenin (GAP)	C_12_H_21_NO_10_S_3_	435.477	[M−H]^-^	434.0254	74.99, **96.96,** 145.05, 195.03, 259.01, 297.23	10, 20, 30	434.02	**97**	28
								259	22
Glucoalyssin (GLS)	C_13_H_25_NO_10_S_3_	451.533	[M−H]^-^	450.0568	91.00 **96.96,** 112.99, 158.98, 174.96, 190.93, 256.96, 259.01, 450.06	10, 20, 30	450.06	**97**	28
								259	22
Glucoiberverin (GBV)	C_11_H_21_NO_9_S_3_	406.048	[M]^-^	406.0306	74.99, 79.96, 95.95, **96.96**, 111.01, 112.01, 164.02, 191.02, 192.02, 195.03, 212.97, 259.01, 274.99, 365.85, 375.99	10, 20, 30	406.03	**97**	28
								259	22
Gluconapoleiferin (GPF)	C_12_H_21_NO_10_S_2_	403.426	[M−H]^-^	402.0534	**96.96,** 112.99, 129.02, 161.05, 174.96, 256.96, 259.01, 306.94	10, 20, 30	402.05	**97**	28
								259	22

**Aromatic GLS**									
Gluconasturtiin (GNS)	C_15_H_21_NO_9_S_2_	423.451	[M−H]^-^	422.0585	74.99, **96.96,** 195.03, 259.01, 358.02	10, 20, 30	422.06	75	43
								**97**	28
								259	22
Glucobarbarin (GBA)	C_15_H_21_NO_10_S_2_	439.450	[M−H]^-^	438.0533	74.99, **96.96,** 135.97, 195.03, 259.01, 332.01	10, 20, 30	438.05	**97**	28
								259	22
Glucotropaeolin (GTR)	C_14_H_19_NO_9_S_2_	409.424	[M−H]^-^	408.0429	74.99,**96.96,** 166.03, 195.03, 259.01	10, 20, 30	408.04	75	43
								**97**	28
								166	25
								195	25
								259	22
Sinalbin (SLB)	C_14_H_19_NO_10_S_2_	425.423	[M−H]^-^	424.0378	74.99, **96.96,** 195.03, 259.01, 360.02	10, 20, 30	424.03	**97**	28
								259	22

**Indole GLS**									
Glucobrassicin (GBR)	C_16_H_20_N_2_O_9_S_2_	448.461	[M−H]^-^	447.0537	74.99, **96.96,** 174.96, 195.03, 205.04, 242.94, 259.01, 274.99	10, 20, 30	447.15	75	43
								**97**	28
								259	22
								275	22
4-Methoxyglucobrassicin (MGB)	C_17_H_22_N_2_O_10_S_2_	478.494	[M−H]^-^	477.0643	74.99, 79.96, **96.96,** 138.97, 195.03, 235.05 259.01, 274.99, 284.00	10, 20, 30	477.06	75	43
								**97**	28
								259	22
								275	19
								292	19
Neoglucobrassicin (NGB)	C_17_H_22_N_2_O_10_S_2_	478.494	[M−H]^-^	477.0643	74.99, 79.96, **96.96,** 154.05, 259.01, 274.99, 290.99, 367.10, 386.06, 446.04	10, 20, 30	477.06	75	43
								**97**	28
								154	22
								259	19
4-hydroxyglucobrassicin (HGB)	C_16_H_20_N_2_O_10_S_2_	464.467	[M−H]^-^	463.0497	74.99, **96.96,** 160.04, 169.04 195.03, 221.04, 259.01, 267.01, 285.02, 383.09	10, 20, 30	463.05	**97**	28
								169	25
								259	22
								275	19
								383	10

**Sulforaphane and *S-*methyl-l-cysteine sulfoxide**									
Sulforaphane (SFN)	C_6_H_11_NOS_2_	177.288	[M + H]^+^	178.0355	55.05, 71.99, **114.04,** 119.05	10, 20, 30	178.00	55	28
								72	20
								**114**	13
Sulforaphane-d8 (SFN-d8)	C_6_H_3_D_8_NOS_2_	185.337	[M + H]^+^	186.0857	62.10, 74.00, **122.09,** 127.10	10, 20, 30	186.30	62	29
								**122**	13
*S*-Methyl-l-cysteine sulfoxide (SMCSO)	C_4_H_9_NO_3_S	151.184	[M + H]^+^	152.0376	70.03, 76.78, **88.04**	10, 20, 30	152.30	42	22
								70	15
								**88**	10
*S*-Methyl-l-cysteine sulfoxide-d3 (SMCSO-d3)	C_4_H_6_D_3_NO_3_S	154.20	[M + H]^+^	155.0564	70.03, **88.04,** 89.04, 109.05	10, 20, 30	155.30	42	24
								70	12
								**88**	10

Abbreviations: GLS, glucosinolates; LC HR-MS, liquid chromatography high-resolution-mass spectrometry; LC-QQQ, liquid chromatography-triple quadrupole mass spectrometry; SNCE, stepped normalised collision energy.

#### 2.3.3 LC QQQ-MS analysis

A Thermo Scientific Ultimate 3000 Liquid Chromatography coupled to a Thermo Scientific TSQ Quantiva Triple Quadrupole mass spectrometer was the unit mass resolution instrument used in this study. Separation of vegetable extracts was achieved on an Xbridge C18 column using LC conditions described in 2.3.2. section and on a BEH Amide column using a mobile phase of water (A) and acetonitrile (B) both containing 10 mM ammonium formate adjusted to pH 3 with formic acid. The gradient separation was completed in 15 min with the initial condition of 100 % solvent B. The linear gradient was as follows: 0–1 min, 100 % B; 1–8 min, 100–60 % B; 8–9 min, 60 % B; 9–10 min 60–100 % B; 10–15 min, 100 % B. The flow rate was 0.4 mL/minute and the column temperature was maintained at 35 °C with an injection volume of 4 µL.

The MS spectra were acquired in multiple reactions monitoring (MRM) mode. Electrospray ionisation in polarity switching mode was applied. The cycle time was set at 0.7 s and the dwell time ranged from 20 to 62 ms for all analytes. The MS conditions were gases (arbitrary units) sheath 35, auxiliary 15, sweep 0; ion transfer temperature 325 °C and vaporizer temperature 275 °C. Nitrogen was used as nebulizer and heater gas and argon was selected as collision gas. The optimal MRM parameters for each analyte including precursor ion, product ion transitions, the base ions (quantifier ions) and collision energies are presented in Table 1.

### 2.4 Sample collection and cooking vegetables samples

Whole broccoli heads (n = 3), Drumhead (white) cabbages (n = 3) and Chinese cabbage (n = 3) were purchased from a local supermarket (Farmer Jacks, North Beach, Western Australia) in September 2021. After washing vegetables, each broccoli was divided into 50 g portions removing only the very woody base of the stem, and cut into uniform, medium bite-size pieces. White cabbages and Chinese cabbages were divided into 100 g and 200 g portions, respectively, and roughly chopped into 5 cm strip widths. One portion of each vegetable was left raw/uncooked and set aside for comparison of before and after cooking. Being such a widely consumed cruciferous, broccoli was chosen to be cooked using 4 different cooking methods including micro-waving, steaming, boiling, and stir-frying, whilst the white and Chinese cabbages were steamed only. For microwaving, the portion was placed into a microwave-proof dish and cooked for exactly 2 min (Convection microwave oven Sharp Carousel; 900 W). For steaming, water was brought to boil, and the portion was placed into a stove-top kitchen steamer (with the lid on) for exactly 3 min. For boiling, the portion/s were simply placed into boiling water (2 L) for exactly 3 min. Lastly, for stir-frying, 3.75 mL of extra-virgin olive oil was added to a pre-heated stir-fry pan and the portion was tossed for exactly 4 min. Following each cooking technique, samples were removed immediately from heat, and let cool at room temperature for approximately 5 min before freezing. All samples were stored in a −80 °C freezer for 24 h, freeze-dried (Sublimate 2, Esco E.U.) and re-weighed before being ground to a fine powder using a coffee grinder (Anko, PCML2012) and stored at −20 °C until analysis.

### 2.5 Sample extraction

Ground raw and cooked cruciferous samples (30 mg) were extracted with 70 % hot methanol (70 °C; 1 mL) and shaken for 20 min at 700 rpm and 70 °C using a microtube Thermal Mix (Thermo Scientific). The extracts were allowed to cool down and centrifuged at 24 °C and 14000 rpm for 10 min. The supernatant (500 µL) was transferred to a separate 1.5 mL Eppendorf tube and diluted v/v (1:0 and 1:40) with 0.1 % aqueous formic acid solution (for Xbridge C18 analysis) or acetonitrile (for BEH Amide analysis). The diluted extracts (990 µL) were then spiked with 10 µL internal standard (ISTD) solution containing GTR, *d3*-SMCSO and *d8*-SFN at 100 µg/mL, vortexed for 2 min and transferred to LC vials for analysis.

#### 2.5.1 Extraction efficiency

The extraction efficiency was estimated by spiking ISTD solution (1 µg/mL) into the ground cruciferous samples (30 mg). The spiked samples were extracted with 70 % hot methanol (1 mL) (as described in 2.5. section). Another set of ground cruciferous samples (30 mg) were extracted with 1 mL of 70 % hot methanol (as described in 2.5. section). The diluted supernatants (1:10 v/v) were spiked with ISTD solution (1 µg/mL). The samples spiked before and after extraction were analysed and extraction efficiency was calculated according to equation (1).

(1) $$\mathrm{Extraction}\;efficiency=\frac{C_{\mathrm{spiked}\;before\;extraction}}{C_{\mathrm{spiked}\;after\;extraction}}\;x\;100$$

### 2.6 Method validation

Method validation was performed on both LC HR-MS (using Xbridge C18 column) and on LC QQQ-MS (using both Xbridge C18 and BEH Amide columns) and in accordance with ICH and IUPAC guidelines (ICH Guideline, 2005; Thompson, Ellison, & Wood, 2002).

#### 2.6.1 Linearity and sensitivity

Linearity was evaluated by constructing calibration curves over the concentration range (0.02–5 µg/mL for GLS, 0.4–100 µg/mL for SMCSO and 0.008–2 µg/mL for SFN. The calibration standards were prepared in 0.1 % aqueous formic acid for analysis using Xbridge C18 column and in acetonitrile for analysis using BEH Amide column. The calibration curves were plotted using the peak area ratio of each analyte to the internal standard (y-axis) versus the concentration (x-axis). Method sensitivity was estimated by calculating the limit of detection (LOD) and the limit of quantification (LOQ) which were determined as signal to noise ratio (S/N) of 3 and 10 of diluted standard solution, respectively (n = 10). Additionally, LOD and LOQ were also calculated using the calibration curve method for the selected analytical method.

#### 2.6.2 Precision, trueness and accuracy

Precision and trueness of the method were determined by an intra-day and inter-day analysis of a set of mix standards (L_1_-L_4_) prepared in 0.1 % aqueous formic acid for analysis using Xbridge C18 column and in acetonitrile for analysis using BEH Amide column. L1 to L4 for MGB, GBN, GBR, GIB, GER, GNA, GNS, GRA, PRO, SIN were 0.12, 0.5, 1.25, and 5 µg/mL; for SFN were 0.048, 0.2, 0.5, and 2 µg/m; and for SMCSO were 2.4, 10, 25 and 100 µg/mL, respectively. The mix standards were injected into Xbridge C18 column and analysed using LC HR-MS six times per day (intra-day) and one time per day for six consecutive days (inter-day). The resulting concentrations of the replicate analysis were used to calculate the coefficient of variation (% CV) and thus precision. The calculated mean concentration relative to the nominal concentration was used to reveal trueness (% bias). The intra-day and inter-day precision and accuracy were also determined for standards (L_1_-L_4_) injected into Xbridge C18 and BEH Amide column and analysed using LC QQQ-MS.

The accuracy of the method was evaluated by recovery test. In brief, aliquots (25 µL) of both pooled broccoli and pooled Chinese cabbage extracts were spiked with mixed standard solutions at 4 different concentrations (L_1_-L_4_) of 0.02, 0.2, 2.5 and 5 µg/mL for GBN, GBR, MGB, GIB, GER, GRA, PRO and SIN; 0.01, 0.1, 1 and 2.5 µg/mL for GNA and GNS; 0.01, 0.1, 0.5 and 1 µg/mL for SFN and 0.5, 2.5, 25 and 50 µg/mL for SMCSO, respectively. The pooled broccoli extract resulted from mixing the extracts from three raw broccoli samples, while the pooled Chinese cabbage extract was prepared by combining the extracts from three raw Chinese cabbage samples. The spiked extracts were then spiked with 10 µL ISTD solution containing GTR, *d3*-SMCSO and *d8*-SFN at 100 µg/mL. The volume of all aliquots was made up to 1000 µL using water, vortexed for 2 min, and transferred to LC vials for analysis. The unspiked pooled broccoli and Chinese cabbage extracts were prepared by adding 10 µL of ISTD solution to 25 µL of pooled extracts, followed by adjusting the volume to 1000 µL with water and vortexing for 2 min. The baseline (unspiked) and spiked extracts at each concentration were analysed on Xbridge C18 column using LC HR-MS and the percentage of recovery was calculated according to equation (2), where *C_baseline_* is the calculated unspiked analyte concentration, *C_recovered_* is the calculated spiked analyte concentration, and *C_spiked_* is the absolute concentration of spiked standard added to the sample.

(2) $$\mathrm{Relative}\;\mathrm{recovery}=\left[\frac{\left(C_{\mathrm{recovered}}-C_{\mathrm{baseline}}\right)}{C_{\mathrm{spiked}}}\right]\;x\;100\%$$

### 2.7 Statistical analysis

Statistical analysis was carried out using the Statistical Package for Social Sciences (SPSS ver. 28, IBM, New York, USA). All data are shown as mean ± standard error and were analysed using one-way analysis of variance (ANOVA). Significant values between data obtained for target analytes in each vegetable extract analysed using different instruments/columns were determined using Tukey HSD post-hoc test and *p* value of < 0.05 was considered significant.

## 3 Results and discussion

### 3.1 Choosing a column and chromatographic conditions for separation

Several columns with different functionalities and mechanisms of separation were tested for their ability to simultaneously retain and resolve 9 intact GLS (GNA, GIB, GER, PRO, GBN, GBR, GRA, SIN, and GTR), SMCSO and SFN. They included two C18 columns (Waters Xbridge C18 and ACE C18), two HILIC columns (Waters ACQUITY UPLC BEH Amide and Thermo Syncronis™ HILIC or Zic HILIC) and a C18 column with embedded PFP functionality (ACE C18-PFP). The C18 and C18-PFP columns were tested using 2 different mobile phases: (1) water: methanol: formic acid; and (2) water: acetonitrile: formic acid. The same gradient was used for all separations involving the C18 and C18-PFP column. It is crucial to highlight that the sensitivity in LC/ESI-MS is predominantly influenced by ionisation efficiency, which greatly varies with the chemical properties of the sample and the composition of the mobile phase. In order to tackle this challenge, formic acid was added to the mobile phase. Formic acid prevents suppression of the electrospray signal and preserves the volatility of the mobile phase. It also functions as an acidifier (pKa 3.74) and a chromatographic ion pairing agent (Cataldi, Rubino, Lelario, & Bufo, 2007).

The ACE C18 column retained and resolved the GLS using either the methanol or acetonitrile containing mobile phase. SFN was also retained and eluted after the GLS. However, SMCSO was not retained and eluted near the dead time (1.3 min; determined both by monitoring the rise in TIC as the unretained species elute and comparing its retention time with that of unretained glucose). Improved retention of SMCSO on a C18 column has previously been achieved using an ion pairing reagent, heptafluorobutyric acid (HFBA), however, the use of ion pairing reagents in conjunction with MS detection has been linked with supressed MS signal and memory effects impacting subsequent analyses and therefore was not explored here (Rütters, Moehring, Rullkötter, Griep-Raming, & Metzger, 2000). Using a C18 column with PFP functionality provided a similar retention pattern to the C18 column, with SMCSO also eluting at the dead time.

Liu et al. (2020) used an Xbridge C18 column to retain SMCSO and therefore this column was investigated. The Xbridge C18 column successfully retained all analytes using either methanol or acetonitrile as the mobile phase. The dead time for this column was just 0.9 min as the flow rate was 0.5 mL/min compared to just 0.2 mL/min flow rate for the ACE C18 and ACE C18-PFP columns. The higher flow rate was necessary to maintain adequate back pressure due to larger, 3 mm, particle size employed in the Xbridge C18 column. Using the Xbridge C18 column and either methanol or acetonitrile as the mobile phase, SMCSO was retained and eluted at 2.2 min (Fig. S1E). A different selectivity was evident with this C18 column when compared to the ACE C18 column, with SMCSO eluting amongst the GLS rather than prior. SFN eluted late as for the ACE C18 column (Fig. S1A). The peak shapes were gaussian and relatively sharp. For the acetonitrile separation theoretical plates in the range 3000–7000 were calculated for GIB, PRO, GRA, SIN and SMCSO, while significantly larger theoretical plates in the range of 60,000–120,000 per column were reported for GNA, GER, GBN, GBR, GTR and SFN. A very different theoretical plates were also reported for the two groups of analytes in methanol. For the methanol separation, the theoretical plates ranged from 60,000 – 90,000 for the GNA, GER, GBN, GBR, GTR and SFN. As expected, the retention time of the later eluting GLS and SFN was increased when methanol replaced acetonitrile as the mobile phase. For example, SFN eluted after 9.5 min in acetonitrile, but after 10.9 min in methanol (Table 2).

**Table 2:** LOD, LOQ and theoretical plates of target analytes analysed using different chromatographic methods.

Instrument	Chromatographic method	Test	MGB	GBN	GBR	GIB	GER	GNA	GNS	GRA	PRO	SIN	GTR	SFN	SMCSO
**LC HR-MS**	**X-bridge/ACN: W (0.1 % FA)**	RT (min)	5.1	4.2	4.5	**1.3**	4.3	3.6	5.0	2.0	1.7	1.9	4.3	9.5	2.2
		LOD (ng/mL)	2.5	0.9	2.1	2.1	1.1	1.3	2.5	3.8	1.8	1.7	1.2	1.0	1.9
		LOQ (ng/mL)	8.4	3.1	7	6.9	3.5	4.4	8.3	12.4	6.1	5.6	4.1	3.1	6.4
		Theoretical Plate	89,355	79,397	71,368	3240	114,877	58,611	119,810	7399	4553	6542	112,760	77,794	4152
	**X-bridge/MeOH: W (0.1 % FA)**	RT (min)	NT#	4.7	5.1	1.4	4.9	3.7	NT	2.1	1.8	1.9	4.8	10.9	2.2
		LOD (ng/mL)	NT	1.1	2.3	2.4	2.5	1.3	NT	3.9	2.3	2.0	1.6	1.0	1.2
		LOQ (ng/mL)	NT	3.5	7.7	8.1	8.2	4.3	NT	12.8	7.6	6.7	5.4	3.2	3.8
		Theoretical Plate	NT	48,951	89,707	2805	68,147	61,578	NT	6594	5803	5792	78,782	66,184	4190
	**ACE PFP (ACN: W 0.1 % FA)**	RT (min)	NT	6.0	6.4	2.0	6.1	5.4	NT	2.5	2.1	2.5	6.1	8.1	1.3
		LOD (ng/mL)	NT	1.7	3.9	2.8	2.5	2.4	NT	3.7	2.1	3.3	2.2	2.2	2.2
		LOQ (ng/mL)	NT	5.6	12.9	9.1	8.3	8.1	NT	12.4	7.0	10.9	7.1	7.3	7.3
		Theoretical Plate	NT	54,481	63,823	3037	56,887	50,943	NT	13,850	4633	4105	56,887	57,074	1824
	**ACE PFP (MeOH: W 0.1 % FA)**	RT (min)	NT	6.6	7.6	2.0	6.9	5.3	NT	2.9	2.3	2.6	6.9	10.1	1.3
		LOD (ng/mL)	NT	2.0	2.7	2.3	2.8	2.2	NT	3.5	2.5	3.2	1.6	1.9	1.1
		LOQ (ng/mL)	NT	6.8	8.8	7.5	9.4	7.4	NT	11.5	6.8	10.7	5.4	6.1	3.5
		Theoretical Plate	NT	43,162	49,867	2976	53,673	16,229	NT	3771	3219	3803	45,005	69,908	2561
	**ACE C18 (ACN: W 0.1 % FA)**	RT (min)	NT	6.0	6.4	1.9	6.1	5.4	NT	2.5	2.2	2.5	6.1	8.1	1.3
		LOD (ng/mL)	NT	1.3	3.6	3.1	1.8	1.7	NT	3.6	3.1	3.3	2.3	1.5	1.9
		LOQ (ng/mL)	NT	4.1	11.9	10.4	5.9	5.5	NT	11.9	10.2	10.9	7.5	4.9	6.1
		Theoretical Plate	NT	55,031	62,247	2392	57,638	44,047	NT	3435	4152	3745	56,700	57,215	2405
	**ACE C18 (MeOH: W 0.1 % FA)**	RT (min)	NT	6.6	7.1	1.8	6.8	5.4	NT	2.9	2.4	2.6	6.7	9.5	1.3
		LOD (ng/mL)	NT	1.4	2.3	2.3	5.1	2.2	NT	2.4	2.3	2.2	2.3	4.7	1.0
		LOQ (ng/mL)	NT	4.6	7.7	7.8	16.7	7.2	NT	8.0	7.6	7.2	7.6	15.5	3.4
		Theoretical Plate	NT	49,399	56,994	2266	49,998	33,091	NT	3303	2877	2773	39,090	68,621	2443
	**Amide HILIC (ACN:W containing 10 mM am. Formate pH 3)**	RT (min)	NT	2.8	3.3	5.6	2.9	3.3	NT	5.5	4.8	3.9	2.7	0.7	6.2
		LOD (ng/mL)	NT	3.0	9.2	5.2	5.0	5.7	NT	9.0	3.0	4.9	5.3	1.0	5.7
		LOQ (ng/mL)	NT	9.9	30.3	17.1	16.5	18.9	NT	29.6	9.8	16.2	17.6	3.4	18.8
		Theoretical Plate	NT	12,300	17,065	189,607	11,063	14,193	NT	266,189	151,142	27,146	11,053	1357	115,546
	**Zic HILIC (ACN:W containing 10 mM am. Formate pH 3)**	RT (min)	NT	2.0	2.3	5.4	2.0	2.5	NT	5.4	4.5	3.2	1.9	0.7	6.5
		LOD (ng/mL)	NT	31.2	28.0	19.1	17.3	24.4	NT	4.1	9.7	9.0	5.0	0.9	29.7
		LOQ (ng/mL)	NT	103.1	92.4	63.0	57.0	80.7	NT	13.6	31.9	29.6	16.5	3.0	98.0
		Theoretical Plate	NT	46	2443	26,947	2306	3222	NT	44,047	15,101	5113	2548	1086	4793
**LC-QQQ**	**X-bridge/ACN: W (0.1 % FA)**	RT (min)	5.0	4.2	4.6	1.4	4.4	3.6	4.9	2.1	1.8	2.0	4.3	9.5	2.2
		LOD (ng/mL)	1.3	1.5	1.3	1.6	1.5	1.2	1.5	1.6	1.8	1.6	1.3	1.7	2.1
		LOQ (ng/mL)	4.1	5.0	4.2	6.0	4.8	3.8	4.9	5.3	5.6	5.2	4.2	5.7	7.0
		Theoretical Plate	28,152	39,651	32,280	4282	42,125	36,049	53,641	3074	3745	8864	28,587	101,609	7448
	**Amide HILIC (ACN:W containing 10 mM am. Formate pH 3)**	RT (min)	3.4	2.9	3.4	5.5	2.9	3.4	2.5	5.5	4.8	4.0	2.8	0.7	6.2
		LOD (ng/mL)	3.0	2.0	5.9	3.9	2.0	6.1	2.3	2.1	4.2	1.8	2.9	5.7	2.8
		LOQ (ng/mL)	9.9	6.7	19.6	12.9	6.5	20.1	7.4	6.8	13.9	5.9	9.6	18.6	9.2
		Theoretical Plate	18,211	12,676	17,999	56,209	19,154	39,791	16,962	54,797	11,577	17,640	14,052	1149	58,774

Abbreviations: MGB, 4-methoxyglucobrassicin; GBN, glucobrassicanapin; GBR, glucobrassicin; GIB, glucoiberin; GER, glucoerucin; GNA, gluconapin; GNS, gluconasturtiin; GRA, glucoraphanin; PRO, progoitrin; SIN, sinigrin; GTR, glucotropaeolin; SFN, sulforaphane; SMCSO, *S*-methyl-l-cysteine sulfoxide; RT, retention time; LOD, limit of detection; LOQ, limit of quantification; MeOH, methanol; ACN; acetonitrile; W, water; FA, formic acid.

# Not Tested.

The analyte mixture was also separated using a BEH Amide and a ZIC column at two pHs (3 and 5) to explore HILIC separations. The separations on the ZIC column were poor as evidenced by poor peak shape (Fig. S1C) and low theoretical plate count (Table 2). The separations on the BEH Amide column, and specifically at pH 3, were characterised by sharp peaks (Fig. S1D) and high theoretical plates (Table 2). Interestingly, the analytes that had lower theoretical plates on the Xbridge C18 column recorded theoretical plates of 110,000–270,000 on the BEH Amide column. However, an important limitation of the BEH Amide column was its inability to retain SFN, which eluted in the dead volume (Fig. S1D). A comparison of LODs for the analytes separated on the BEH Amide column with the Xbridge C18 column, also reported LODs typically 3 times higher (Table 2). LOD and LOQ values, calculated using the calibration curve method for the analysis with an Xbridge C18 column on an LC HR-MS, are reported in Table S4.

In summary, an Xbridge C18 column was selected for this study as it effectively resolved and retained the three different classes of compounds using an acetonitrile, water, and formic acid mobile phase. The BEH Amide column was competitive with respect to peak shape and LODs for all analytes, but it was ineffective at retaining SFN. However, for separations where SFN is not of interest, it is an excellent choice for SMCSO and GLS measurements. It is also useful for validation of results produced on the C18 column as the mechanism of separation is different. Therefore, in this work, the BEH Amide column was used to validate the results obtained on the Xbridge C18 column.

**Fig. 1:**
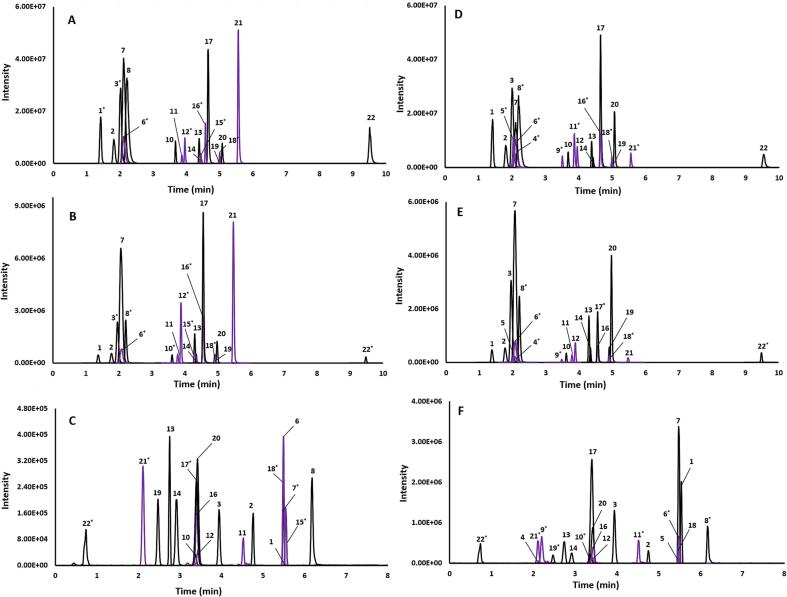
Overlay of extracted ion chromatograms of (1) glucoiberin; (2) progoitrin, (3) sinigrin, (4) glucoraphenin, (5) glucocheirolin, (6) glucobarbarin, (7) glucoraphanin, (8) S-methyl-l-cysteine sulfoxide, (9) gluconapoleiferin, (10) gluconapin, (11) 4-hydroxyglucobrassicin, (12) glucoibervierin, (13) glucotropaeolin, (14) glucobrassicin, (15) Sinalbin, (16) glucoalyssin, (17) glucobrassicin, (18) glucoberteroin, (19) gluconasturtiin, (20) 4-methoxyglucobrassicin, (21) neoglucobrassicin, (22) sulforaphane in (A) raw broccoli extract separated on Xbridge C18 column acquired on liquid chromatography high-resolution-mass spectrometry (LC HR-MS), (B) raw broccoli extract separated on Xbridge C18 column and acquired on liquid chromatography-triple quadrupole mass spectrometry (LC QQQ-MS), (C) raw broccoli extract separated on BEH Amide column and acquired on LC QQQ-MS, (D) raw white cabbage extract separated on Xbridge C18 column and acquired on LC HR-MS, (E) raw white cabbage extract separated on Xbridge C18 column and acquired on LC QQQ-MS, and (F) raw white cabbage extract separated on BEH Amide column and acquired on LC QQQ-MS. *The peak intensities have been updated to fit within the scales of chromatograms.

Two further standards, MGB and GNS were received after completion of this method development. MGB eluted at 5.1 min on the Xbridge column (Fig. 1A, B, D and E) and at 3.4 min on the BEH Amide column (Fig. 1C and F). GNS eluted at 5 min on the Xbridge column (Fig. 1A, B, D and E) and 2.5 min on the Amide column (Fig. 1C and F).

### 3.2 Identification of GLS in vegetable extracts

In addition to the 11 GLS standards (GNA, GIB, GER, PRO, GBN, GBR, GRA, SIN, MGB, GNS and GTR) selected in this study, a review of the literature identified several other GLS of interest present in cruciferous vegetables (Hooshmand et al., 2021; Hwang et al., 2019, Liang et al., 2018). Therefore, vegetable extracts of broccoli, white cabbage, and Chinese cabbage were analysed using LC HR-MS by further scanning for the molecular ions of other potential GLS. The MS/MS pattern associated with these molecular ions were compared with MS/MS patterns reported in the literature for GLS. Furthermore, we verified that the assigned tentative GLS had previously been reported in the cruciferous vegetables (Table S1). For example, in this study glucobarbarin (GBA) was detected in broccoli, which is supported by Liang et al. (2018) who confirmed its presence in a number of brassica vegetables using a certified standard. The tentatively identified GLS through this process include: glucocheirolin (GOC), glucoberteroin (GOB), glucoraphenin (GAP), glucobarbarin (GBA), sinalbin (SLB), 4-hydroxyglucobrassicin (HGB), glucoalyssin (GLS), glucoiberverin (GBV), gluconapoleiferin (GPF) and neoglucobrassicin (NGB) (Table 1 and Table S1).

GNS shares the same mass number as tentatively identified GIB (423), and they are easily distinguishable on a high-resolution instrument due to their unique fragmentation pattern (Table 1). These isobars (of mass 423) are chromatographically resolved on both the Xbridge C18 and BEH Amide columns (Fig. 1A and B), making their analysis on a unit resolution instrument (QQQ-MS) possible (Fig. 1B, C, E and F). MGB and its tentatively identified isomer NGB were also chromatographically fully resolved from each other on the Xbridge C18 column (Fig. 1A, B, D and E) and baseline separated on the BEH Amide column (Fig. 1C and F). Two of the tentatively identified GLS and GBA (439.450) GOC (439.465) are isobars. Again, while easily distinguished using high resolution mass spectrometry, chromatographic separation is required for unit resolution MS instruments. Both these analytes are fully resolved on the Xbridge C18 (Fig. 1D and E) and BEH Amide columns (Fig. 1F). The LC HR-MS method developed here can identify 21 GLS, SFN and SMCSO, and chromatographically resolve isobars and isomers, making the method transferrable to a unit resolution instrument. The method was also used to analyse vegetable extracts to confirm peak shape and sensitivity were maintained in real extracts (Fig. 1). The chemical structures of all 21 GLS, SFN, and SMCSO are presented in Fig. S4.

### 3.3 Calibration standards

While standards were available for 11 GLS, there are no standards currently available for the additional 10 GLS that were tentatively identified using high resolution MS. Quantitation of intact GLS using a single GLS standard or a representative GLS standard for each class of GLS has been previously reported, (Bello et al., 2018, Liang et al., 2018, Mocniak et al., 2023) but this approach assumes the ionisation efficiency is similar and that any suppression or enhancement of signal is consistent for the analyte GLS and surrogate standard. To test the validity of such approach, the detector response (or ionisation efficiency) for a 1 µg/mL standard mixture of 11 GLS was measured on two different instruments (LC QQQ-MS and LC HR-MS), at two time points (several months apart) on the LC HR-MS and using two different column/mobile phases. For each experiment, the peak area of each analyte (precursor ion for the LC HR-MS and base ion for the LC QQQ-MS) was normalised to the analyte having the lowest detector response (Table 3). The GLS recorded varying ionisation efficiencies. For separations on the Xbridge column ionisation efficiencies varied as much as 3-fold for the GLS, while using the BEH Amide column and the same MS detection method there was as much as a 30-fold difference in detector response between the different GLS. The differences in detector response between columns are to be expected as different mobile phases will impact ionisation efficiency. For the Xbridge C18 column on either LC QQQ-MS or LC HR-MS, the fold difference in detector response between analytes was as much as 3, with GRA providing the greatest detector response on the LC QQQ-MS and SIN the greatest response on the LC HR-MS. The difference in detector response between instruments is also not unexpected – the LC HR-MS uses the molecular ion for quantitation while the LC QQQ-MS uses the base ion for quantitation.

**Table 3:** Detector response calculated by diving the peak area of each glucosinolate (1 µg/mL) by the glucosinolate with the lowest peak area.

Glucosinolates	LC HR-MS Xbridge C18 column*	LC HR-MS Xbridge C18 column**	LC QQQ-MS Xbridge C18 column*	LC QQQ-MS BEH Amide column*
**4-Methoxyglucobrassicin (MGB)**	1.1	1.6	1.9	10.9
**Glucobrassicin (GBR)**	1.0	1.0	1.6	10.0
**Glucobrassicanapin (GBN)**	2.4	3.0	2.6	24.8
**Glucoiberin (GIB)**	2.2	2.5	1.5	1.0
**Glucoerucin (GER)**	2.2	2.1	2.4	29.6
**Gluconapin (GNA)**	1.9	2.3	1.4	1.6
**Glucoraphanin (GRA)**	1.4	1.6	3.1	6.8
**Progoitrin (PRO)**	2.0	2.6	1.0	3.6
**Sinigrin (SIN)**	3.2	3.1	1.5	10.5
**Gluconasturtiin (GNS)**	2.0	2.3	2.8	22.9
**Glucotropaeolin (GTR)**	1.7	1.8	2.6	19.1

Abbreviations: LC HR-MS, liquid chromatography high-resolution-mass spectrometry; LC QQQ-MS, liquid chromatography-triple quadrupole mass spectrometry.

* Calculate from mean of 6 injections on a single day.

** Calculated from mean of 6 injections on a single day, 9 months later.

For a given separation, consistent ionisation was not always evident even within the GLS class. For the aliphatic GLS, there was as much as a 10-fold difference in detector response using the BEH Amide column, a 3-fold difference in detector response using the Xbridge C18 column, and up to a 2-fold difference in detector response using the LC HR-MS over time. Therefore, the use of surrogate standards to quantify the GLS is not informative and must be avoided.

Quantitative analysis by LC-MS, is generally achieved using the method of internal standard calibration. The internal standard is ideally the isotopically labelled standard. Labelled standards are available for SFN and SMCSO, however, they are not readily available for GLS, or are expensive. Sinigrin, a GLS present in vegetables, and usually at relatively low concentrations in broccoli, has been used as the internal standard. However, for broader *Brassica* vegetable studies where SIN contribution can be significant (e.g., kale and cauliflower), an alternative internal standard is required (Hwang et al., 2019). More suitable internal standards are GTR or SLB which are present in very low concentrations (<1 % for cruciferous vegetables studied here) as shown by Hwang et al. (2019). The background levels of both GTR and SLB were checked in a subset of samples by calculating their percentage contribution in the samples. The percentage contribution of GTR and SLB was calculated by dividing the peak area of GTR and SLB detected in each sample by the total area of all GLS detected in that sample and multiplying by 100. The results demonstrated that both GTR and SLB contributions are < 1 % of the area of total GLS for the cruciferous vegetables studied here (Table S2). Considering GTR availability, it was chosen as the ISTD for GLS analysis in this study.

### 3.4 Extraction method

Intact GLS are typically extracted from dried plant material using hot aqueous methanol (Hooshmand et al., 2021; Hwang et al., 2019, Liang et al., 2018). The elevated temperature is required to stop myrosinase-mediated hydrolysis of the intact GLS (Hooshmand et al., 2021). Several extraction methods have been reported for the extraction of SMCSO, most notably aqueous methanol with o-(carboxymethyl)hydroxylamine hemihydrochloride added to inhibit deconjugation of SMCSO by the enzyme alliinase (Bernaert et al., 2012). Hot methanol has also been reported as an effective extraction method for SMCSO (Friedrich et al., 2022). SFN is polar, making aqueous methanol an appropriate extraction solvent. Therefore, 70 % hot (70 °C) methanol was used to extract the GLS, SMCSO and SFN from dried, ground vegetable material.

Extractions involving 10, 20, 30, 40 and 50 mg of dried vegetable material extracted into 1 mL of 70 % hot methanol for 20 min were completed. The extracts were diluted 1 in 50 and the peak areas for each analyte recorded for the different extracts. There was a linear increase in the detector response for all analytes (r^2^ > 0.97) with increase in mass of vegetable material extracted (Figs. S2 and S3). An extraction mass of 30 mg and a dilution volume of 10 or 40 (to avoid some analytes exceeding the calibration range) were used for the remaining studies. Peaks were gaussian and the peak areas recorded were within the linear range of the calibration curves. The extraction efficiency was determined, with efficiencies of greater than 80 % recorded for SMCSO and the GLS, and 78 % for SFN for the different vegetable samples (Table S3).

### 3.5 Method validation

The linear calibration range for each of the calibration standards was first determined experimentally on the LC HR-MS. SMCSO which is present in high concentrations showed excellent linearity up to 100 µg/mL (r^2^ = 0.999). Cruciferous extracts diluted 1 in 10 typically recorded SMCSO concentrations in the range 20–80 µg/mL and therefore within the calibration range. The linear calibration range for the GLS was 0.02–5 µg/mL with r^2^ values of 0.999 for MGB, GBN, GBR, GIB, GNS, GRA, and PRO and 0.998 for GER, GNA, and SIN. Higher concentrations showed a non-linear response. Using the 30 mg/mL of 70 % hot methanol and a 1 in 10 dilution had most of the GLS fall within the calibration range, with a couple of important exceptions: the concentration of GBR in broccoli and white cabbage exceeded the calibration range when the extract was diluted 1 in 10, so a 1 in 40 dilution is more appropriate; and the concentration of MGB in Chinese cabbage also exceeded the calibration range, so a 1 in 20 or 1 in 40 dilution is more appropriate. SFN is present in low concentrations, and a 1 in 10 dilution was ample for all samples tested. Given the natural variability of analytes in vegetables, and the impact that different treatments or variables may have on the analytes of interest, the relationship between sample extracts and calibration range needs to be checked and the dilution modified as appropriate.

The intra-day and inter-day accuracy (% bias) and precision (% CV) were determined by analysing four different concentrations of calibration standards for six replicates in a single day (intra-day) and one replicate for six consecutive days (inter-day). The bias for both intra-day and inter-day studies ranged from − 17 to 15 %. The CV ranged from 1 to 18 % (Table S5). The relative recovery in broccoli and Chinese cabbage extracts ranged from 80 to 120 % which indicates accuracy of the method (Table S6).

### 3.6 Routine analysis

For large studies involving many samples, access to a HR-MS is not always possible, so the feasibility of transferring the LC HR-MS method to a LC QQQ-MS system was tested. The linear calibration range reported on the LC HR-MS was successfully repeated on the LC QQQ-MS. The robustness of the quantitative and qualitative data generated by the LC QQQ-MS method was tested by generating and comparing experimental data from both the LC QQQ-MS and the LC HR-MS. Experimental data was also collected on two columns employing different mechanisms of separation – HILIC (BEH Amide column) and reversed phase (Xbridge C18 column). Nine vegetable samples (in triplicate) were analysed. A statistical investigation of the data found that there was no statistical difference in the concentration reported for any of the analytes measured on either the column or instrument used (Table 4). Additionally, both the column and instrument successfully detected the presence of the 10 tentatively identified GLS. Remarkably, there was a perfect 100 % concordance between the LC QQQ-MS and LC HR-MS, as well as the Xbridge C18 and BEH Amide columns (Table 5). This method has been successfully applied to monitor impact of different cooking methods on GLS and SMCSO concentrations in different cruciferous vegetables (manuscript in preparation).

**Table 4:** Mean ± Std. Errors (mg/g), mean Square Errors and *p* values obtained for target analytes in each vegetable extract analysed on different instruments; liquid chromatography high-resolution-mass spectrometer (LC HR-MS) and liquid chromatography-triple quadrupole mass spectrometer (LC QQQ-MS) and different columns; Xbridge C18 and BEH Amide using a one-way ANOVA and Tukey HSD post-hoc analyses.

Analyte	Comparison	Broccoli raw	Broccoli steamed	Broccoli boiled	Broccoli microwaved	Broccoli stir-fried	White cabbage raw	White cabbage steamed	Chinese cabbage raw	Chinese cabbage steamed
**MGB**	Mean ± Std. Error (mg/g; N = 9)	0.30 ± 0.020	0.52 ± 0.025	0.30 ± 0.012	0.46 ± 0.032	0.23 ± 0.019	0.82 ± 0.10	0.76 ± 0.068	1.9 ± 0.33	1.8 ± 0.35
	Mean Square Error (between groups)	0.00093	0.0054	0.0010	0.019	0.0023	0.017	0.0018	0.20	0.32
	*p* value	0.82	0.43	0.49	0.11	0.55	0.87	0.97	0.86	0.80
**GBN**	Mean ± Std. Error (mg/g; N = 9)	ND*	ND	ND	ND	ND	BLQ	BLQ	0.92 ± 0.17	0.82 ± 0.15
	Mean Square Error (between groups)	NA**	NA	NA	NA	NA	NA	NA	0.0015	0.00067
	*p* value	NA	NA	NA	NA	NA	NA	NA	1.0	1.0
**GBR**	Mean ± Std. Error (mg/g; N = 9)	3.9 ± 0.12	4.1 ± 0.17	1.6 ± 0.14	3.8 ± 0.33	2.4 ± 0.45	4.0 ± 0.17	4.3 ± 0.22	0.42 ± 0.094	0.46 ± 0.10
	Mean Square Error (between groups)	0.38	0.015	0.26	0.84	1.3	0.43	0.53	0.018	0.014
	*p* value	0.051	0.96	0.28	0.49	0.57	0.22	0.14	0.84	0.88
**GIB**	Mean ± Std. Error (mg/g; N = 9)	0.97 ± 0.090	0.82 ± 0.13	0.48 ± 0.019	0.79 ± 0.033	0.48 ± 0.063	3.8 ± 0.18	4.1 ± 0.31	ND	ND
	Mean Square Error (between groups)	0.063	0.11	0.0024	0.014	0.012	0.97	1.4	NA	NA
	*p* value	0.48	0.54	0.53	0.27	0.78	0.79	0.096	NA	NA
**GER**	Mean ± Std. Error (mg/g; N = 9)	0.081 ± 0.011	0.15 ± 0.038	0.099 ± 0.015	0.14 ± 0.016	0.051 ± 0.0097	0.082 ± 0.015	0.080 ± 0.015	0.021 ± 0.0019	0.029 ± 0.0032
	Mean Square Error (between groups)	0.00061	0.0039	0.0019	0.0030	0.0010	0.00028	0.00030	0.000054	0.000011
	*p* value	0.63	0.78	0.43	0.23	0.28	0.77	0.87	0.15	0.91
**GNA**	Mean ± Std. Error (mg/g; N = 9)	0.27 ± 0.033	0.29 ± 0.055	0.17 ± 0.015	0.28 ± 0.025	0.14 ± 0.020	0.21 ± 0.027	0.16 ± 0.025	1.5 ± 0.40	1.5 ± 0.45
	Mean Square Error (between groups)	0.0014	0.000047	0.00026	0.0010	0.000062	0.0026	0.0021	0.32	0.29
	*p* value	0.90	1.0	0.90	0.86	0.99	0.71	0.72	0.83	0.87
**GNS**	Mean ± Std. Error (mg/g; N = 9)	0.065 ± 0.010	0.085 ± 0.0072	0.046 ± 0.0050	0.074 ± 0.0076	0.026 ± 0.0049	0.12 ± 0.018	0.14 ± 0.019	0.42 ± 0.017	0.41 ± 0.015
	Mean Square Error (between groups)	0.00032	0.00032	0.00017	0.00019	0.00017	0.0013	0.00031	0.0088	0.0035
	*p* value	0.75	0.53	0.45	0.71	0.43	0.69	0.93	0.11	0.21
**GRA**	Mean ± Std. Error (mg/g; N = 9)	4.1 ± 0.30	4.9 ± 0.36	1.9 ± 0.18	3.9 ± 0.25	2.1 ± 0.16	1.8 ± 0.086	2.0 ± 0.096	0.056 ± 0.0027	0.058 ± 0.0060
	Mean Square Error (between groups)	0.59	0.97	0.057	0.56	0.041	0.086	0.090	0.000059	0.000010
	*p* value	0.55	0.50	0.87	0.41	0.87	0.31	0.21	0.48	0.98
**PRO**	Mean ± Std. Error (mg/g; N = 9)	0.45 ± 0.048	0.77 ± 0.13	0.23 ± 0.022	0.46 ± 0.027	0.24 ± 0.027	0.53 ± 0.037	0.47 ± 0.074	0.89 ± 0.16	0.87 ± 0.16
	Mean Square Error (between groups)	0.022	0.0075	0.0011	0.0079	0.0031	0.0031	0.0016	0.0079	0.0064
	*p* value	0.40	0.96	0.82	0.35	0.69	0.82	0.97	0.98	0.98
**SIN**	Mean ± Std. Error (mg/g; N = 9)	0.19 ± 0.022	0.18 ± 0.029	0.12 ± 0.014	0.19 ± 0.013	0.098 ± 0.014	1.9 ± 0.16	1.7 ± 0.037	ND	ND
	Mean Square Error (between groups)	0.0013	0.00049	0.00059	0.0017	0.00012	0.0039	0.019	NA	NA
	*p* value	0.78	0.95	0.76	0.40	0.95	0.99	0.12	NA	NA
**SFN**	Mean ± Std. Error (mg/g; N = 9)	0.013 ± 0.00046	NT#	NT	NT	NT	0.0051 ± 0.00041	NT	ND	NT
	Mean Square Error (between groups)	0.0000037	NT	NT	NT	NT	0.000000037	NT	NA	NT
	*p* value	0.13	NT	NT	NT	NT	0.98	NT	NA	NT
**SMCSO**	Mean ± Std. Error (mg/g; N = 9)	13 ± 0.63	13 ± 0.75	6.6 ± 0.29	13 ± 0.88	8.9 ± 0.42	7.9 ± 0.34	5.6 ± 0.68	4.6 ± 0.25	4.2 ± 0.29
	Mean Square Error (between groups)	1.00	1.8	0.11	1.0	0.54	0.031	0.18	0.16	0.00058
	*p* value	0.81	0.75	0.90	0.89	0.77	0.98	0.97	0.81	1.0

Abbreviations: MGB, 4-methoxyglucobrassicin; GBN, glucobrassicanapin; GBR, glucobrassicin; GIB, glucoiberin; GER, glucoerucin; GNA, gluconapin; GNS, gluconasturtiin; GRA, glucoraphanin; PRO, progoitrin; SIN, sinigrin; SFN, sulforaphane; SMCSO, and S-methyl-l-cysteine sulfoxide.

# Not Tested.

* Not detected.

** Not applicable.

**Table 5:** Tentative identification of glucosinolates without calibration standards in raw, steamed, boiled, microwaved and stir-fried broccoli; raw, and steamed white cabbage; and raw and steamed Chinese cabbage analysed on different instruments; liquid chromatography high-resolution-mass spectrometer (LC HR-MS) and liquid chromatography-triple quadrupole mass spectrometer (LC QQQ-MS) and different columns; Xbridge C18 and BEH Amide.

Sample	Instrument	Column	GOC	GOB	GAP	GBA	SLB	HGB	GLS	GBV	GPF	NGB
**Broccoli raw**	**LC HR-MS**	**Xbridge C18**	**–**	√	**–**	√	√	√	√	√	**–**	√
	**LC QQQ-MS**	**Xbridge C18**	**–**	√	**–**	√	√	√	√	√	**–**	√
		**BEH Amide**	**–**	√	**–**	√	√	√	√	√	**–**	√
**Broccoli steamed**	**LC HR-MS**	**Xbridge C18**	**–**	√	**–**	√	√	√	√	√	**–**	√
	**LC QQQ-MS**	**Xbridge C18**	**–**	√	**–**	√	√	√	√	√	**–**	√
		**BEH Amide**	**–**	√	**–**	√	√	√	√	√	**–**	√
**Broccoli boiled**	**LC HR-MS**	**Xbridge C18**	**–**	√	**–**	√	√	√	√	√	**–**	√
	**LC QQQ-MS**	**Xbridge C18**	**–**	√	**–**	√	√	√	√	√	**–**	√
		**BEH Amide**	**–**	√	**–**	√	√	√	√	√	**–**	√
**Broccoli microwaved**	**LC HR-MS**	**Xbridge C18**	**–**	√	**–**	√	√	√	√	√	**–**	√
	**LC QQQ-MS**	**Xbridge C18**	**–**	√	**–**	√	√	√	√	√	**–**	√
		**BEH Amide**	**–**	√	**–**	√	√	√	√	√	**–**	√
**Broccoli stir-fried**	**LC HR-MS**	**Xbridge C18**	**–**	√	**–**	√	√	√	√	√	**–**	√
	**LC QQQ-MS**	**Xbridge C18**	**–**	√	**–**	√	√	√	√	√	**–**	√
		**BEH Amide**	**–**	√	**–**	√	√	√	√	√	**–**	√
**White cabbage raw**	**LC HR-MS**	**Xbridge C18**	√	√	√	√	**–**	√	√	√	√	√
	**LC QQQ-MS**	**Xbridge C18**	√	√	√	√	**–**	√	√	√	√	√
		**BEH Amide**	√	√	√	√	**–**	√	√	√	√	√
**White cabbage steamed**	**LC HR-MS**	**Xbridge C18**	√	√	√	√	**–**	√	√	√	√	√
	**LC QQQ-MS**	**Xbridge C18**	√	√	√	√	**–**	√	√	√	√	√
		**BEH Amide**	√	√	√	√	**–**	√	√	√	√	√
**Chinese cabbage raw**	**LC HR-MS**	**Xbridge C18**	**–**	√	√	**–**	√	√	√	**–**	√	√
	**LC QQQ-MS**	**Xbridge C18**	**–**	√	√	**–**	√	√	√	**–**	√	√
		**BEH Amide**	**–**	√	√	**–**	√	√	√	**–**	√	√
**Chinese cabbage steamed**	**LC HR-MS**	**Xbridge C18**	**–**	√	√	**–**	√	√	√	**–**	√	√
	**LC QQQ-MS**	**Xbridge C18**	**–**	√	√	**–**	√	√	√	**–**	√	√
		**BEH Amide**	**–**	√	√	**–**	√	√	√	**–**	√	√

Abbreviations: GOC, glucocheirolin; GOB, glucoberteroin; GAP, glucoraphenin; SLB, sinalbin; HGB, 4-hydroxyglucobrassicin; GLS, glucoalyssin; GBV, glucoiberverin; GPF, gluconapoleiferin; and NGB, neoglucobrassicin.

## 4 Conclusion

In the present study, we successfully developed an analytical method using HR-MS. We extended the applicability of the method to include QQQ-MS detection, making the method more amenable to large studies involving many samples. The method quantified 10 GLS, SMCSO and SFN and provided qualitative data for another 10 tentatively assigned GLS. The method was successfully applied to different cruciferous vegetables. The applicability of the method to monitor changes in GLS and SMCSO due to various cooking techniques was a key driver behind the development of this method and was well demonstrated. Typically, previous studies have analysed SMCSO and GLS separately, and few studies have included SFN analysis in vegetables as a measure of freshness (Bello et al., 2018, Campas-Baypoli et al., 2010). The development and optimisation of this method enable the accurate and efficient quantification of GLS, SMCSO and SFN levels in commonly consumed cruciferous vegetables. The accurate measurement of these compounds will enhance our understanding of how various cooking methods impact concentrations of these important compounds found in cruciferous vegetables and expand our knowledge to other plant foods. Importantly, this knowledge will aid nutrition and health researchers in future human studies to explore potential dose-dependent health effects of these compounds, ultimately to inform optimal dietary intake requirements.

## CRediT authorship contribution statement

**Armaghan Shafaei:** Conceptualization, Methodology, Validation, Formal analysis, Resources, Writing – original draft, Writing – review & editing, Visualization. **Caroline R. Hill:** Data curation, Methodology, Resources. **Jonathan M. Hodgson:** Conceptualization, Methodology, Supervision. **Lauren C. Blekkenhorst:** Conceptualization, Methodology, Supervision. **Mary C. Boyce:** Conceptualization, Methodology, Supervision, Writing – review & editing.

## Declaration of competing interest

The authors declare that they have no known competing financial interests or personal relationships that could have appeared to influence the work reported in this paper.

## Data Availability

Data will be made available on request.
