# Supplementary material for: Simultaneous extraction and quantitative analysis of S-Methyl-l-Cysteine Sulfoxide, sulforaphane and glucosinolates in cruciferous vegetables by liquid chromatography mass spectrometry
Source: Food Chem X. 2023 Dec 14;21:101065. doi: 10.1016/j.fochx.2023.101065 (PMC10767375; doi:10.1016/j.fochx.2023.101065)
Supplement: Supplementary data 1 [file mmc1.docx]

# Simultaneous Extraction and Quantitative Analysis of *S*-Methyl-L-Cysteine Sulfoxide, Sulforaphane and Glucosinolates in Cruciferous Vegetables by Liquid Chromatography Mass Spectrometry

Armaghan Shafaei^a*^, Caroline R. Hill^b^, Jonathan M. Hodgson^b,c^, Lauren C. Blekkenhorst^b,c^, and Mary C. Boyce^d^

^a^ Centre for Integrative Metabolomics & Computational Biology, School of Science, Edith Cowan University, Joondalup, Western Australia, Australia

^b^ Nutrition and Health Innovation Research Institute, School of Medical and Health Sciences, Edith Cowan University, Perth, Western Australia, Australia

^c^ Royal Perth Hospital Research Foundation, Perth, Australia

^d^ School of Science, Edith Cowan University, Joondalup, Western Australia, Australia

^*^ Corresponding Author.

E-mail: [a.shafaeidarestani@ecu.edu.au](mailto:a.shafaeidarestani@ecu.edu.au)

**Table S1.** Comparison of the tentatively identified glucosinolates in this study with literature. **Base ions** are highlighted in bold.

| **Tentative identification** | **Vegetable(s) in this study** | **Vegetable(s) in literature** | **Fragments reported in literature** | **Fragments identified in this study (LC HR-MS)** | **Fragments applied in this study (LC QQQ-MS)** |
| --- | --- | --- | --- | --- | --- |
| **Glucocheirolin (GOC)** | White cabbage | Broccoli (Maldini, Baima, Morelli, Scaccini, & Natella, 2012)  Cabbage (Liang, Lee, Li, Lu, Zou, & Ong, 2018) | 358  97, 259 | 74.99, **96.96,** 135.97, 195.03, 259.01, 332.01 | **97,** 259 |
| **Glucoberteroin (GOB)** | Broccoli, white cabbage and Chinese cabbage | Chinese cabbage (Yu, Ma, Zhang, & Li, 2020)    Kimchi cabbage and radish (Hwang, Park, Dang, Kim, & Seo, 2019) | 354.11 258.98 195.03^*^  354.11, 259.01, 195.03, 128.93, 96.96, 74.99 | 74.99, **96.96,** 119.04, 128.93, 195.03, 214.00, 259.01, 274.99 | **97,** 129, 195, 259, 354 |
| **Glucoraphenin (GAP)** | White cabbage and Chinese cabbage | Kale (Hwang et al., 2019)  Red radish and radish (Liang et al., 2018)  Daikon radish and daikon radish leaves (Yu et al., 2020) | 297.23, 259.01, 195.03, 145.05, 96.96, 74.99  97, 259  96.50 258.98 419.01 | 74.99, **96.96,** 145.05, 195.03, 259.01, 297.23 | **97**, 259 |
| **Glucobarbarin (GBA)** | Broccoli and white cabbage | Kale and mustard (Hwang et al., 2019)  Rocket salad, radish, red radish, pakchoi, choy sum, Kai Lan, broccoli, cabbage, Brussels sprouts and Chinese cabbage (Liang et al., 2018) | 332.01, 259.01, 195.03, 135.97, 96.96, 74.99  97, 259 | 74.99, **96.96,** 135.97, 195.03, 259.01, 332.01 | **97**, 259 |
| **Sinalbin (SLB)** | Broccoli and Chinese cabbage | Broccoli, cabbage and kale, kimchi cabbage, mustard, and young radish root (Hwang et al., 2019)  Used as internal standard (Glauser, Schweizer, Turlings, & Reymond, 2012)  Chemical standard was studied (Rochfort, Trenerry, Imsic, Panozzo, & Jones, 2008) | 360.02, 259.01, 195.03, 96.96, 74.99  259, 182, 96  344, 291, 275, 261, 259, 246, 231, 228, 182 | 74.99, **96.96,** 195.03, 259.01, 360.02 | **97**, 259 |
| **4-hydroxyglucobrassicin (HGB)** | Broccoli, white cabbage and Chinese cabbage | Rocket, watercress, red radish, radish, pakchoi, Chinese cabbage, choy sum, Brussels sprouts, cabbage, cauliflower, broccoli, Kai Lan (Liang et al., 2018)  Broccoli sprouts (Maldini et al., 2012)  Mustard seed, broccoli seed, Brassica sprouts (Rochfort et al., 2008)  Broccoli, rocket, Chinese cabbage, pakchoi, cauliflower, baby Chinese cabbage, Chinese kale, cabbage, Daikon radish, Daikon radish leaves (Yu et al., 2020) | 97, 259  267^*^  383, 285, 275, 267, 259, 240  285.00, 267.00, 259.01 | 74.99, **96.96,** 160.04, 169.04, 195.03, 221.04, 259.01, 267.01, 285.02, 383.09 | **97,** 169, 259, 275, 383 |
| **Glucoalyssin (GLS)** | Broccoli, white cabbage and Chinese cabbage | Rocket, watercress, red radish, radish, pakchoi, Chinese cabbage, choy sum, Brussels sprouts, broccoli, kai Lan (Liang et al., 2018)  Broccoli sprouts (Maldini et al., 2012)  Red cabbage (Wu, Chen, Yu, Chen, Ye, & Zhang, 2021) | 97, 259  386^*^  97^*^ | 91.00 **96.96,** 112.99, 158.98, 174.96, 190.93, 256.96, 259.01, 450.06 | **97**, 259 |
| **Gluconapoleiferin (GPF)** | Chinese cabbage | Broccoli sprouts (Maldini et al., 2012)  Pakchoi, Chinese cabbage and choy sum (Liang et al., 2018)  Chinese cabbage (Yu et al., 2020)  Rapeseed (Millán et al., 2009) | 195^*^  97, 259^*^  332.00, 258.98, 195.00^*^  135.6, 194.7, 209.6, 225.5, 258.7, 274.6, 307.8, 331.7^#^ | **96.96,** 112.99, 129.02, 161.05, 174.96, 256.96, 259.01, 306.94 | 97, 259 |
| **Neoglucobrassicin (NGB)** | Broccoli, white cabbage and Chinese cabbage | Broccoli sprouts (Maldini et al., 2012)  Brassica sprouts (Rochfort et al., 2008)  Radish, pakchoi, Chinese cabbage, choy sum, Brussels sprouts, cabbage, cauliflower, and kai Lan (Liang et al., 2018)  Red cabbage (Wu et al., 2021)  Broccoli, rocket, Chinese cabbage, pakchoi, cauliflower, baby Chinese cabbage, Chinese kale, cabbage, Daikon radish, Daikon radish leaves (Yu et al., 2020)  Chemical standard was studied (Fabre et al., 2007) | 446^*^  447, 466, 284, 259  97, 259^*^  97, 239.1, 305.1, 422.1^*^  96.96 258.98 446.00  447, 446, 259, 416, 285, 284, 275, 251, 241, 224, 201, 195, 188 | 74.99, 79.96, **96.96,** 154.05, 259.01, 274.99, 290.99, 367.10, 386.06, 446.04 | 75, **97,** 154, 259 |
| **Glucoiberverin (GBV)** | Broccoli, white cabbage and Chinese cabbage | Broccoli seed, brassica sprouts (Rochfort et al., 2008)  Mustard seeds, leaves and roots (Al-Gendy, Nematallah, Zaghloul, & Ayoub, 2016) | 326, 275, 259, 228, 145, 195^*^  275, 259, 228^*^ | 74.99, 79.96, 95.95, **96.96**, 111.01, 112.01, 164.02, 191.02, 192.02, 195.03, 212.97, 259.01, 274.99, 365.85, 375.99 | **97**, 259 |

* Without chemical standard

^#^ Certified reference material was used

**Table S2.** Percentage contribution of glucotropaeolin (GTR) and sinalbin (SLB) in different cruciferous extracts

| **Samples** | **%Contribution**  **GTR** | **%Contribution**  **SLB** |
| --- | --- | --- |
| **Raw broccoli** | 0.05 | 0.02 |
| **Raw white cabbage** | 0.03 | ND^*^ |
| **Raw Chinese cabbage** | 0.07 | < 0.001 |

**^*^**Not detected

**Table S3.** Extraction efficiency of internal standards in different cruciferous extracts

| **Samples** | **%Extraction Recovery**  **(SMCSO-d3)** | **Extraction %RSD**  **(SMCSO-d3)** | **%Extraction Recovery**  **(SFN-d8)** | **Extraction %RSD**  **(SFN-d8)** | **%Extraction Recovery**  **(GTR)** | **Extraction %RSD**  **(GTR)** |
| --- | --- | --- | --- | --- | --- | --- |
| **Raw broccoli** | 86.60 | 4.55 | 78.09 | 6.11 | 95.99 | 6.20 |
| **Raw white cabbage** | 87.78 | 0.91 | 81.45 | 2.01 | 99.00 | 4.58 |
| **Raw Chinese cabbage** | 89.51 | 1.86 | 79.25 | 5.20 | 84.84 | 10.77 |

**Table S4.** LOD and LOQ of target analytes calculated using the calibration curve method and analysed using an Xbridge C18 column on an LC HR-MS instrument

| **Analyte** | **LOD**  **(ng/mL)** | **LOQ**  **(ng/mL)** |
| --- | --- | --- |
| **MGB** | 28.71 | 87.01 |
| **GBN** | 35.57 | 107.77 |
| **GBR** | 16.46 | 49.87 |
| **GIB** | 43.54 | 131.93 |
| **GER** | 30.65 | 92.89 |
| **GNA** | 30.90 | 93.64 |
| **GNS** | 9.92 | 30.06 |
| **GRA** | 48.38 | 146.60 |
| **PRO** | 41.54 | 125.89 |
| **SIN** | 46.51 | 140.94 |
| **SFN** | 7.16 | 21.70 |
| **SMCSO** | 516.37 | 1564.76 |

**Table S5.** Intra-day and inter-day precision and accuracy.

|  |  | **Analyte** | **Intra-day precision** | | | |  | **Intra-day accuracy** | | | |  | **Inter-day precision** | | | |  | **Inter-day accuracy** | | | | |
| --- | --- | --- | --- | --- | --- | --- | --- | --- | --- | --- | --- | --- | --- | --- | --- | --- | --- | --- | --- | --- | --- | --- |
|  |  |  | **(% CV, n=6)** | | | |  | **(% bias, n=6)** | | | |  | **(% CV, n=6)** | | | |  | **(% bias, n=6)** | | | | |
|  |  |  | **L1^*^** | **L2^*^** | **L3^*^** | **L4^*^** |  | **L1^*^** | **L2^*^** | **L3^*^** | **L4^*^** |  | **L1^*^** | **L2^*^** | **L3^*^** | **L4^*^** |  | **L1^*^** | **L2^*^** | **L3^*^** | **L4^*^** | |
| **LC HR-MS Xbridge C18 column** |  | **MGB** | 3.38 | 1.83 | 2.21 | 2.90 |  | 4.03 | 4.43 | -2.41 | 2.33 |  | 2.10 | 1.50 | 1.72 | 4.09 |  | 1.81 | 1.73 | -2.79 | -2.06 |  |
|  |  | **GBN** | 2.96 | 3.87 | 3.47 | 3.20 |  | 4.31 | 10.77 | 6.17 | 0.51 |  | 2.88 | 2.22 | 3.39 | 3.87 |  | 5.00 | 7.27 | 7.73 | -1.51 |  |
|  |  | **GBR** | 4.17 | 3.03 | 3.19 | 2.03 |  | -0.97 | 4.20 | -0.59 | -0.52 |  | 2.49 | 2.39 | 3.05 | 2.07 |  | -1.25 | 0.20 | -1.33 | 0.59 |  |
|  |  | **GIB** | 5.04 | 3.74 | 0.87 | 3.20 |  | -14.86 | 4.83 | 1.17 | 0.08 |  | 4.51 | 1.40 | 3.41 | 3.19 |  | -10.56 | 5.93 | 2.28 | -0.38 |  |
|  |  | **GER** | 5.18 | 1.64 | 2.95 | 2.04 |  | -0.14 | 9.20 | 7.81 | -0.34 |  | 8.85 | 1.84 | 4.77 | 2.42 |  | -1.25 | 1.73 | 2.08 | -0.35 |  |
|  |  | **GNA** | 5.13 | 2.96 | 2.32 | 3.61 |  | -16.11 | 4.27 | 2.53 | -1.07 |  | 4.90 | 1.70 | 3.32 | 5.33 |  | -12.22 | 6.47 | 1.57 | 0.73 |  |
|  |  | **GNS** | 3.83 | 1.06 | 3.86 | 3.68 |  | -3.06 | 4.00 | -0.56 | 1.59 |  | 3.62 | 1.72 | 2.75 | 4.00 |  | -3.33 | 2.33 | -0.96 | -1.56 |  |
|  |  | **GRA** | 4.96 | 3.91 | 2.43 | 1.88 |  | -11.94 | 4.20 | 0.47 | -0.09 |  | 1.58 | 0.79 | 4.33 | 2.68 |  | -9.31 | 5.60 | 1.97 | -0.14 |  |
|  |  | **PRO** | 6.71 | 2.79 | 2.29 | 3.11 |  | -9.31 | 4.93 | -0.31 | -0.10 |  | 1.84 | 1.69 | 3.77 | 1.88 |  | -6.25 | 5.17 | 1.35 | -0.05 |  |
|  |  | **SIN** | 5.16 | 4.82 | 2.24 | 2.73 |  | -11.81 | 5.90 | -0.05 | -0.83 |  | 2.11 | 2.13 | 2.87 | 1.91 |  | -7.78 | 4.97 | 2.39 | 0.59 |  |
|  |  | **SFN** | 2.38 | 1.57 | 1.23 | 1.66 |  | 2.43 | 0.50 | 2.80 | 0.56 |  | 1.69 | 3.29 | 10.60 | 1.41 |  | 3.12 | 2.50 | -4.67 | -0.54 |  |
|  |  | **SMCSO** | 0.96 | 1.12 | 1.20 | 1.90 |  | -1.75 | -1.47 | -3.05 | 4.10 |  | 2.48 | 1.08 | 1.33 | 0.42 |  | -0.15 | -0.51 | -1.63 | 5.27 |  |
| **LC QQQ-MS Xbridge C18 column** |  | **MGB** | 10.86 | 4.10 | 14.62 | 4.72 |  | -3.33 | 4.70 | 0.07 | -2.24 |  | 3.56 | 4.58 | 4.71 | 2.13 |  | -4.17 | 6.03 | 3.41 | -0.58 | |
|  |  | **GBN** | 7.41 | 2.42 | 4.63 | 5.21 |  | -12.22 | 3.07 | 5.36 | -3.04 |  | 5.28 | 9.51 | 4.24 | 3.31 |  | -10.83 | -12.00 | 6.67 | 2.26 | |
|  |  | **GBR** | 4.44 | 2.64 | 2.60 | 3.30 |  | -17.22 | -0.57 | 3.87 | 0.23 |  | 5.39 | 4.41 | 1.79 | 4.10 |  | -12.36 | 3.37 | 4.36 | -0.75 | |
|  |  | **GIB** | 1.41 | 3.90 | 3.76 | 1.91 |  | 24.17 | -1.20 | -4.23 | -0.39 |  | 4.29 | 4.33 | 2.72 | 2.58 |  | 9.31 | -2.77 | -6.77 | 1.08 | |
|  |  | **GER** | 5.47 | 3.62 | 4.27 | 4.75 |  | 0.42 | 3.47 | 2.24 | 1.57 |  | 2.74 | 5.72 | 3.48 | 3.07 |  | -3.19 | 0.13 | -2.15 | -1.60 | |
|  |  | **GNA** | 3.77 | 2.15 | 3.50 | 1.93 |  | -10.83 | 0.67 | 5.45 | -1.76 |  | 3.42 | 3.11 | 3.55 | 2.63 |  | -10.69 | 5.67 | 7.05 | 0.95 | |
|  |  | **GNS** | 9.23 | 4.55 | 10.81 | 2.71 |  | -10.69 | 3.57 | 2.87 | 0.45 |  | 5.46 | 4.32 | 4.12 | 3.53 |  | -11.81 | 5.97 | 5.35 | -1.02 | |
|  |  | **GRA** | 4.03 | 3.11 | 3.63 | 1.61 |  | -11.81 | -2.30 | 1.52 | -2.01 |  | 1.75 | 3.81 | 3.37 | 3.81 |  | -11.39 | 1.80 | 3.65 | 1.70 | |
|  |  | **PRO** | 2.70 | 4.18 | 4.95 | 1.85 |  | -5.69 | -2.47 | 1.17 | -0.42 |  | 4.45 | 6.51 | 3.57 | 3.31 |  | -6.67 | -1.73 | 1.63 | 1.05 | |
|  |  | **SIN** | 3.80 | 4.04 | 3.73 | 1.45 |  | -9.44 | -1.63 | 0.84 | -1.61 |  | 1.77 | 4.33 | 2.94 | 2.76 |  | -8.47 | 2.50 | 2.68 | 1.40 | |
|  |  | **SFN** | 4.92 | 3.67 | 3.89 | 5.01 |  | 14.93 | -0.83 | -4.87 | 1.29 |  | 5.77 | 2.16 | 2.35 | 1.04 |  | 12.85 | -0.75 | -1.87 | -0.88 | |
|  |  | **SMCSO** | 5.99 | 5.04 | 4.42 | 4.89 |  | -16.84 | 2.96 | 7.50 | 0.65 |  | 9.55 | 8.87 | 6.08 | 3.33 |  | -13.48 | -3.24 | 1.79 | -1.20 | |
| **LC QQQ-MS BEH Amide column** |  | **MGB** | 3.29 | 3.47 | 1.75 | 0.92 |  | 6.32 | 7.21 | 10.17 | -1.27 |  | 2.63 | 4.14 | 2.50 | 1.40 |  | 5.70 | 1.58 | 7.92 | 0.11 | |
|  |  | **GBN** | 10.33 | 1.06 | 2.66 | 1.11 |  | 14.86 | 5.43 | 7.39 | -3.31 |  | 15.75 | 2.69 | 2.35 | 2.46 |  | 14.72 | 5.20 | 12.33 | 2.02 | |
|  |  | **GBR** | 11.46 | 2.95 | 1.64 | 0.77 |  | 16.25 | 7.71 | 10.65 | -1.96 |  | 13.12 | 3.98 | 3.35 | 3.54 |  | 6.81 | 3.39 | 10.26 | 0.58 | |
|  |  | **GIB** | 6.06 | 5.59 | 7.51 | 4.71 |  | -0.10 | -2.11 | 1.24 | -2.20 |  | 3.74 | 5.18 | 2.73 | 2.33 |  | -5.52 | -5.50 | 0.63 | 2.21 | |
|  |  | **GER** | 13.89 | 0.59 | 1.81 | 1.64 |  | 17.36 | 8.96 | 10.25 | -3.24 |  | 6.82 | 1.98 | 2.88 | 2.43 |  | 10.69 | 9.22 | 13.81 | 1.62 | |
|  |  | **GNA** | 5.12 | 5.40 | 1.98 | 2.05 |  | 6.59 | -3.62 | -11.92 | 0.14 |  | 7.20 | 5.66 | 4.16 | 5.19 |  | 9.74 | -4.60 | -11.46 | 1.45 | |
|  |  | **GNS** | 6.91 | 2.22 | 1.13 | 1.75 |  | 10.52 | 8.54 | 7.96 | -2.21 |  | 5.62 | 2.88 | 2.75 | 2.52 |  | 7.34 | 6.26 | 11.34 | 0.91 | |
|  |  | **GRA** | 5.12 | 7.36 | 5.23 | 6.42 |  | 6.74 | -5.05 | -5.88 | -3.62 |  | 4.52 | 6.57 | 4.32 | 3.58 |  | 8.27 | -8.11 | -6.33 | 4.53 | |
|  |  | **PRO** | 5.14 | 6.22 | 6.02 | 4.27 |  | -5.41 | -6.15 | 1.77 | -3.58 |  | 9.26 | 5.97 | 8.45 | 6.07 |  | -4.60 | -5.32 | 4.88 | 3.29 | |
|  |  | **SIN** | 5.55 | 1.97 | 3.78 | 3.22 |  | 8.95 | 3.13 | 18.10 | -2.79 |  | 9.09 | 4.57 | 2.93 | 2.01 |  | 11.14 | 1.78 | 8.37 | 0.58 | |
|  |  | **SFN** | 12.59 | 12.39 | 17.51 | 15.80 |  | -2.82 | 0.02 | -10.68 | 2.94 |  | 9.78 | 7.68 | 12.99 | 14.44 |  | 5.73 | -3.49 | -7.72 | -1.80 | |
|  |  | **SMCSO** | 13.16 | 7.71 | 5.46 | 12.63 |  | 12.29 | 9.22 | 1.39 | -2.94 |  | 10.39 | 5.29 | 5.77 | 12.57 |  | 9.48 | 9.78 | 1.64 | 5.46 | |

^*^L1 to L4 for MGB, GBN, GBR, GIB, GER, GNA, GNS, GRA, PRO, and SIN were 0.12, 0.5, 1.25, and 5 µg/mL; for SFN were 0.048, 0.2, 0.5, and 2 µg/mL; and for SMCSO were 2.4, 10, 25 and 100 µg/mL, respectively.

**Table S6.** Relative recovery of glucosinolates, sulforaphane, and S-methyl-L-cysteine sulfoxide in raw broccoli and Chinese cabbage extracts separated on Xbridge C18 column and acquired on LC HR-MS.

| **Compound** | **Concentration in broccoli extract before spiking**  **(µg/mL ± SD)** | **Relative recovery** | | | | | | | | | | | | | | | | | | |
| --- | --- | --- | --- | --- | --- | --- | --- | --- | --- | --- | --- | --- | --- | --- | --- | --- | --- | --- | --- | --- |
|  |  | **L1^*^** | | |  | **L2^*^** | | | |  | | **L3^*^** | |  | **L4^*^** | | | | | |
|  |  | **Concentration after spiking (µg/mL ± SD)** | **Recovery (%)** |  |  | **Concentration after spiking (µg/mL ± SD)** | **Recovery (%)** |  |  | | **Concentration after spiking (nM ± SD)** | | **Recovery (%)** |  |  | | **Concentration after spiking (nM ± SD)** | | **Recovery (%)** |  |
| **MGB** | 2.84 ± 0.09 | 2.86 ± 0.00 | 95.00 |  |  | 3.02 ± 0.00 | 91.17 |  |  | | 5.12 ± 0.03 | | 91.08 |  |  | | 7.52 ± 0.26 | | 93.59 |  |
| **GBN** | ND | 0.02 ± 0.00 | 119.67 |  |  | 0.18 ± 0.01 | 90.50 |  |  | | 2.33 ± 0.05 | | 93.21 |  |  | | 4.35 ± 0.07 | | 87.04 |  |
| **GBR** | 1.62 ± 0.04 | 1.65 ± 0.00 | 120.00 |  |  | 1.86 ± 0.04 | 120.67 |  |  | | 4.65 ± 0.21 | | 121.17 |  |  | | 6.57 ± 0.19 | | 99.03 |  |
| **GLB** | 0.26 ± 0.00 | 0.28 ± 0.00 | 83.33 |  |  | 0.50 ± 0.00 | 120.17 |  |  | | 2.56 ± 0.03 | | 92.08 |  |  | | 4.74 ± 0.15 | | 89.59 |  |
| **GLC** | 0.02 ± 0.00 | 0.05 ± 0.01 | 113.33 |  |  | 0.27 ± 0.01 | 121.00 |  |  | | 2.37 ± 0.04 | | 93.92 |  |  | | 4.67 ± 0.12 | | 92.95 |  |
| **GNA** | 0.03 ± 0.00 | 0.05 ± 0.00 | 120.00 |  |  | 0.15 ± 0.00 | 117.00 |  |  | | 0.88 ± 0.02 | | 84.57 |  |  | | 2.43 ± 0.14 | | 96.03 |  |
| **GNS** | 0.03 ± 0.00 | 0.04 ± 0.00 | 106.67 |  |  | 0.15 ± 0.00 | 120.33 |  |  | | 0.94 ± 0.03 | | 90.73 |  |  | | 2.78 ± 0.05 | | 109.91 |  |
| **GRA** | 1.26 ± 0.04 | 1.29 ± 0.00 | 111.67 |  |  | 1.45 ± 0.04 | 91.67 |  |  | | 3.42 ± 0.05 | | 86.25 |  |  | | 5.62 ± 0.16 | | 87.06 |  |
| **PRO** | 0.23 ± 0.00 | 0.25 ± 0.00 | 111.67 |  |  | 0.46 ± 0.02 | 113.17 |  |  | | 2.49 ± 0.05 | | 90.25 |  |  | | 4.48 ± 0.16 | | 85.04 |  |
| **SIN** | 0.05 ± 0.00 | 0.07 ± 0.00 | 106.67 |  |  | 0.29 ± 0.01 | 119.00 |  |  | | 2.60 ± 0.03 | | 101.97 |  |  | | 4.63 ± 0.20 | | 91.52 |  |
| **SFN** | 0.01 ± 0.00 | 0.02 ± 0.00 | 106.67 |  |  | 0.12 ± 0.00 | 106.67 |  |  | | 0.54 ± 0.01 | | 104.67 |  |  | | 0.99 ± 0.01 | | 97.43 |  |
| **SMCSO** | 6.73 ± 0.05 | 7.22 ± 0.08 | 96.73 |  |  | 9.59 ± 0.15 | 114.43 |  |  | | 30.91 v 0.47 | | 96.72 |  |  | | 54.74 ± 0.25 | | 96.04 |  |
| **Compound** | **Concentration in Chinese cabbage extract before spiking**  **(µg/mL ± SD)** | **Relative recovery** | | | | | | | | | | | | | | | | | | |
|  |  | **L1** | | |  | **L2** | | |  | | **L3** | | | | |  | | **L4** | | |
|  |  | **Concentration after spiking (µg/mL ± SD)** | **Recovery (%)** |  |  | **Concentration after spiking (µg/mL ± SD)** | **Recovery (%)** |  |  | | **Concentration after spiking (nM ± SD)** | | **Recovery (%)** |  |  | | **Concentration after spiking (nM ± SD)** | | **Recovery (%)** |  |
| **MGB** | 1.83 ± 0.06 | 1.85 ± 0.00 | 116.67 |  |  | 2.01 ± 0.01 | 92.33 |  |  | | 4.35 ± 0.10 | | 100.76 |  |  | | 5.84 ± 0.29 | | 80.11 |  |
| **GBN** | 0.34 ± 0.01 | 0.36 ± 0.00 | 113.33 |  |  | 0.57 ± 0.01 | 114.50 |  |  | | 2.67 ± 0.02 | | 93.41 |  |  | | 4.81 ± 0.18 | | 89.53 |  |
| **GBR** | 0.60 ± 0.01 | 0.62 ± 0.01 | 93.33 |  |  | 0.79 ± 0.01 | 96.33 |  |  | | 3.48 ± 0.08 | | 115.32 |  |  | | 5.63 ± 0.05 | | 100.71 |  |
| **GLB** | ND | 0.02 ± 0.00 | 110.00 |  |  | 0.21 ± 0.01 | 105.00 |  |  | | 2.35 ± 0.11 | | 94.05 |  |  | | 4.31 ± 0.04 | | 86.18 |  |
| **GLC** | ND | 0.02 ± 0.00 | 101.67 |  |  | 0.21 ± 0.01 | 106.17 |  |  | | 2.48 ± 0.02 | | 99.09 |  |  | | 4.42 ± 0.09 | | 88.49 |  |
| **GNA** | 0.26 ± 0.01 | 0.27 ± 0.00 | 120.00 |  |  | 0.37 ± 0.01 | 110.00 |  |  | | 1.16 ± 0.15 | | 90.10 |  |  | | 2.51 ± 0.06 | | 90.21 |  |
| **GNS** | 0.27 ± 0.01 | 0.28 ± 0.00 | 100.00 |  |  | 0.37 ± 0.01 | 99.00 |  |  | | 1.27 ± 0.09 | | 100.54 |  |  | | 3.04 ± 0.02 | | 110.99 |  |
| **GRA** | 0.02 ± 0.00 | 0.05 ± 0.00 | 113.33 |  |  | 0.26 ± 0.01 | 118.17 |  |  | | 2.41 ± 0.10 | | 95.40 |  |  | | 4.41 ± 0.06 | | 87.71 |  |
| **PRO** | 0.21 ± 0.01 | 0.23 ± 0.01 | 91.67 |  |  | 0.43 ± 0.00 | 109.00 |  |  | | 2.52 ± 0.07 | | 92.21 |  |  | | 4.37 ± 0.10 | | 83.19 |  |
| **SIN** | ND | 0.02 ± 0.00 | 118.00 |  |  | 0.21 ± 0.01 | 104.83 |  |  | | 2.66 ± 0.10 | | 106.49 |  |  | | 4.38 ± 0.14 | | 87.51 |  |
| **SFN** | ND | 0.01 ± 0.00 | 106.67 |  |  | 0.11 ± 0.00 | 107.67 |  |  | | 0.51 ± 0.01 | | 102.67 |  |  | | 0.98 ± 0.01 | | 97.73 |  |
| **SMCSO** | 5.68 ± 0.04 | 6.23 ± 0.10 | 110.40 |  |  | 8.51 ± 0.06 | 113.03 |  |  | | 29.66 ± 0.30 | | 95.93 |  |  | | 53.65 ± 0.11 | | 95.93 |  |

^*^L1 to L4 for GBN, GBR, MGB, GIB, GER, GRA, PRO and SIN were of 0.02, 0.2, 2.5 and 5 µg/mL; for GNA and GNS were 0.01, 0.1, 1 and 2.5 µg/mL; for SFN were 0.01, 0.1, 0.5 and 1 µg/mL; and for SMCSO were 0.5, 2.5, 25 and 50 µg/mL, respectively.

**Matrix effect**

To evaluate potential matrix effects, and in the absence of a stripped sample extract (sample matrix without target analytes), the following approach was adopted: three pooled broccoli, white cabbage and Chinese cabbage samples were prepared from raw and cooked samples and the concentrations of target analytes (GLS, SFN and SMCSO) were determined. Solutions of target analytes of similar concentrations to these samples (n = 3) were then prepared in water with 0.1 % formic acid (no attempt to matrix match). The sample extracts and solutions containing target analytes were then both spiked with 5 μL of L4 (see the accuracy of the method in section 2.6.2) and the detector response was compared for both, and the percentage matrix effect (matrix ion suppression/enhancement) was calculated using equation (1), where R_matrix_ is the response of the given analyte in matrix and R_standard_ is the response in standard solution.

$$\%ME=\frac{R_{matrix}- R_{standard}}{R_{standard}} \times100\% (1)$$

If ME ∼ 0% there is no observed matrix effect, and if ME > 0% then an ion-enhancement occurred, and if ME < 0% an ion-suppression occurred.

**Table S7.** Effect of matrix on ion suppression and ion enhancement (n = 3)

| **Analyte** | **Matrix effect in broccoli extract (%)** | **Matrix effect in white cabbage extract (%)** | **Matrix effect in Chinese cabbage extract (%)** |
| --- | --- | --- | --- |
| **MGB** | -1.3 | -2.3 | -1.9 |
| **GBN** | -2.1 | -1.4 | -2.3 |
| **GBR** | 0.94 | -2.7 | -3.7 |
| **GLB** | 3.6 | -3.1 | 0.65 |
| **GLC** | -3.2 | -1.4 | 0.99 |
| **GNA** | -2.1 | 0.87 | -1.6 |
| **GNS** | -1.9 | 1.23 | -2.2 |
| **GRA** | -2.1 | -1.2 | -3.1 |
| **PRO** | -3.8 | -3.2 | -1.4 |
| **SIN** | -2.5 | 0.64 | 1.0 |
| **SFN** | 1.8 | -0.54 | -0.96 |
| **SMCSO** | -0.91 | 1.1 | 0.77 |


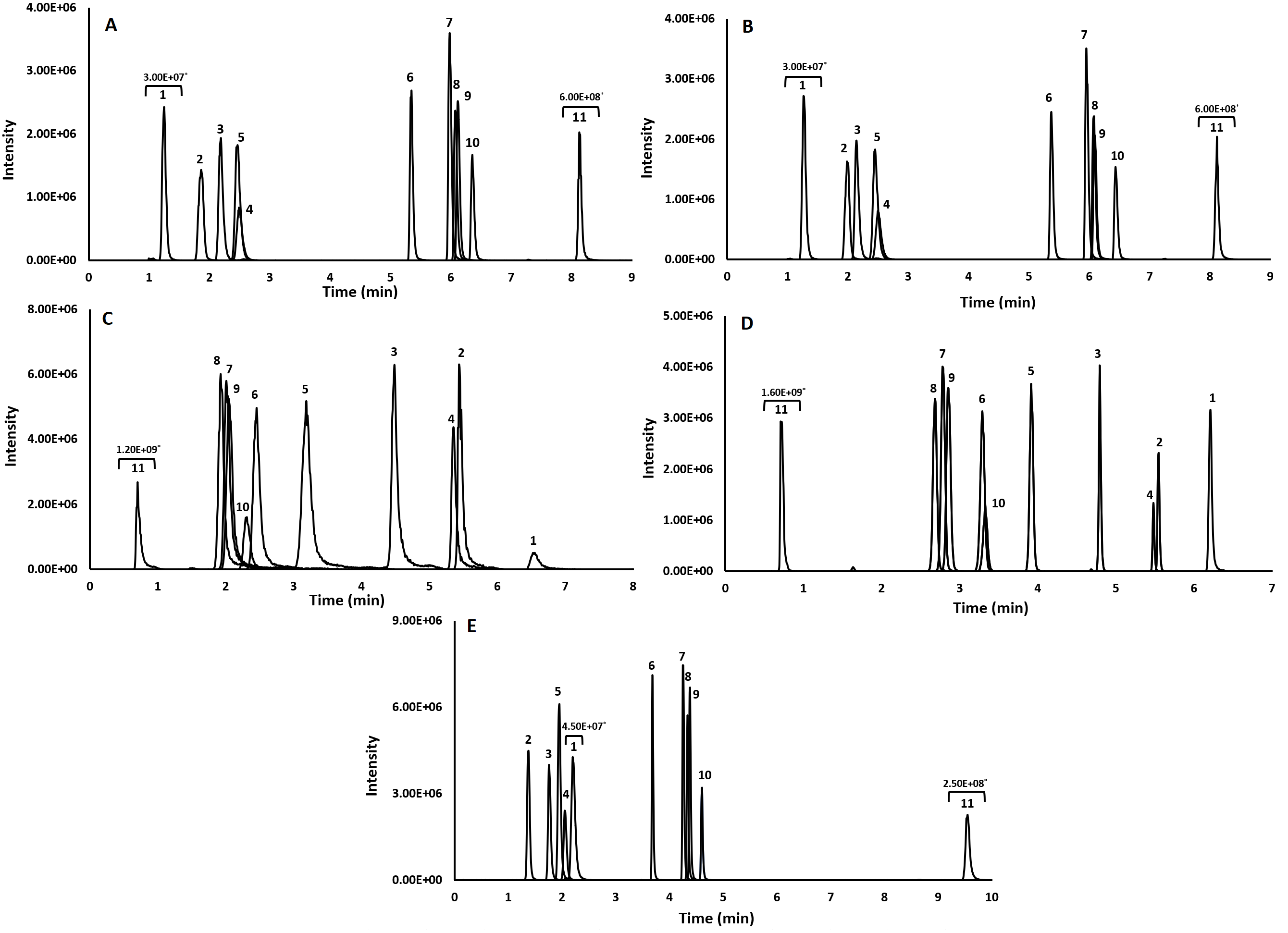


**Fig. S1.** Overlay of extracted ion chromatograms of (1) *S*-methyl-L-cysteine sulfoxide, (2) glucoiberin, (3) progoitrin, (4) glucoraphanin, (5) sinigrin, (6) gluconapin, (7) glucobrassicanapin, (8) glucotropaeolin, (9) glucoerucin, (10) glucobrassicin, and (11) sulforaphane standards separated on (A) ACE C18 column and ACN, (B) ACE C18 PFP column, (C) Thermo Zic HILIC column, (D) BEH Amide column, and (E) and Xbridge C18 column acquired on liquid chromatography high-resolution-mass spectrometry (LC HR-MS) in full scan-MS^2^ mode. ^*^Refers to peak intensity.


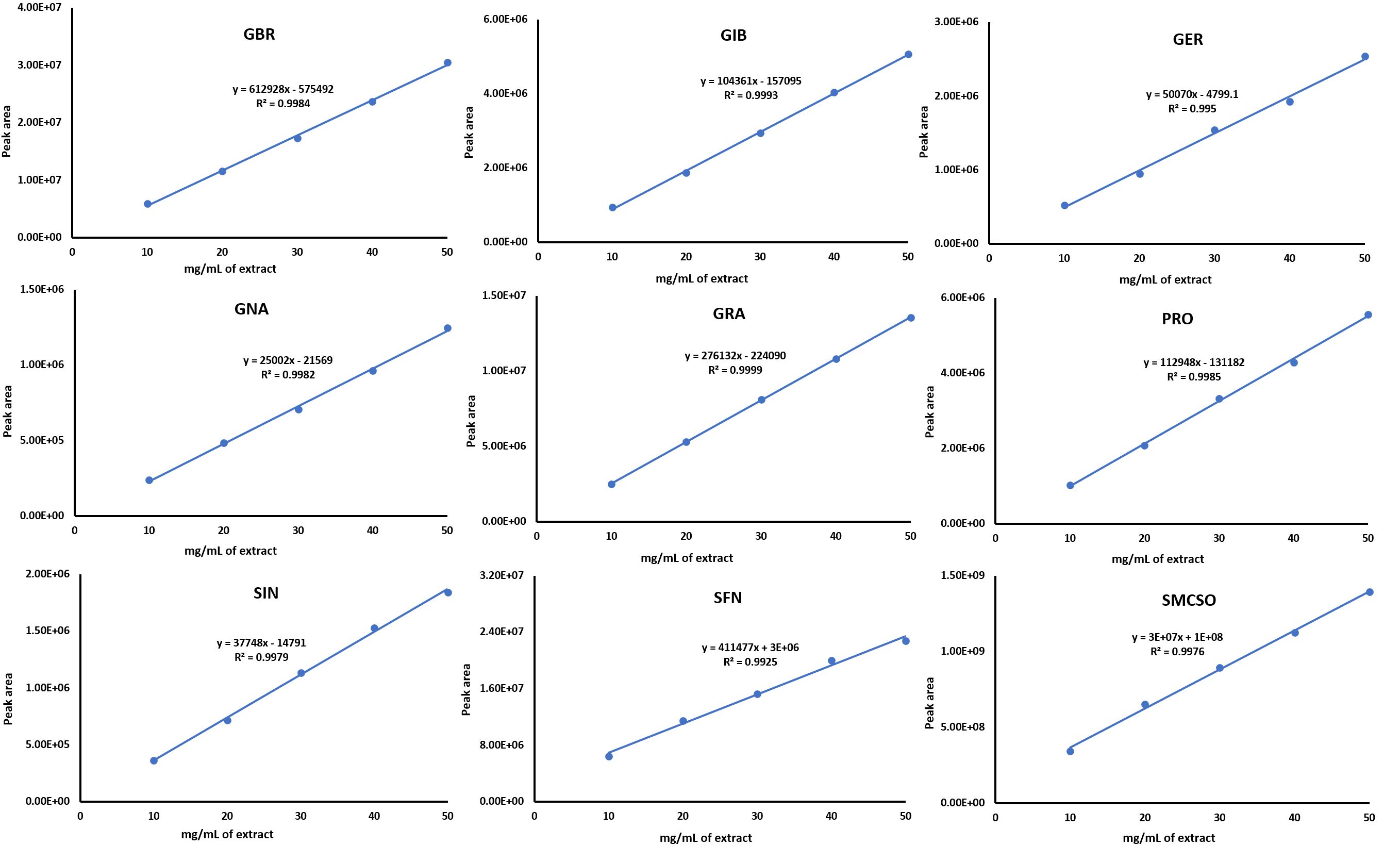


**Fig. S2.** Test the linearity of target analytes at different extraction concentrations in broccoli.


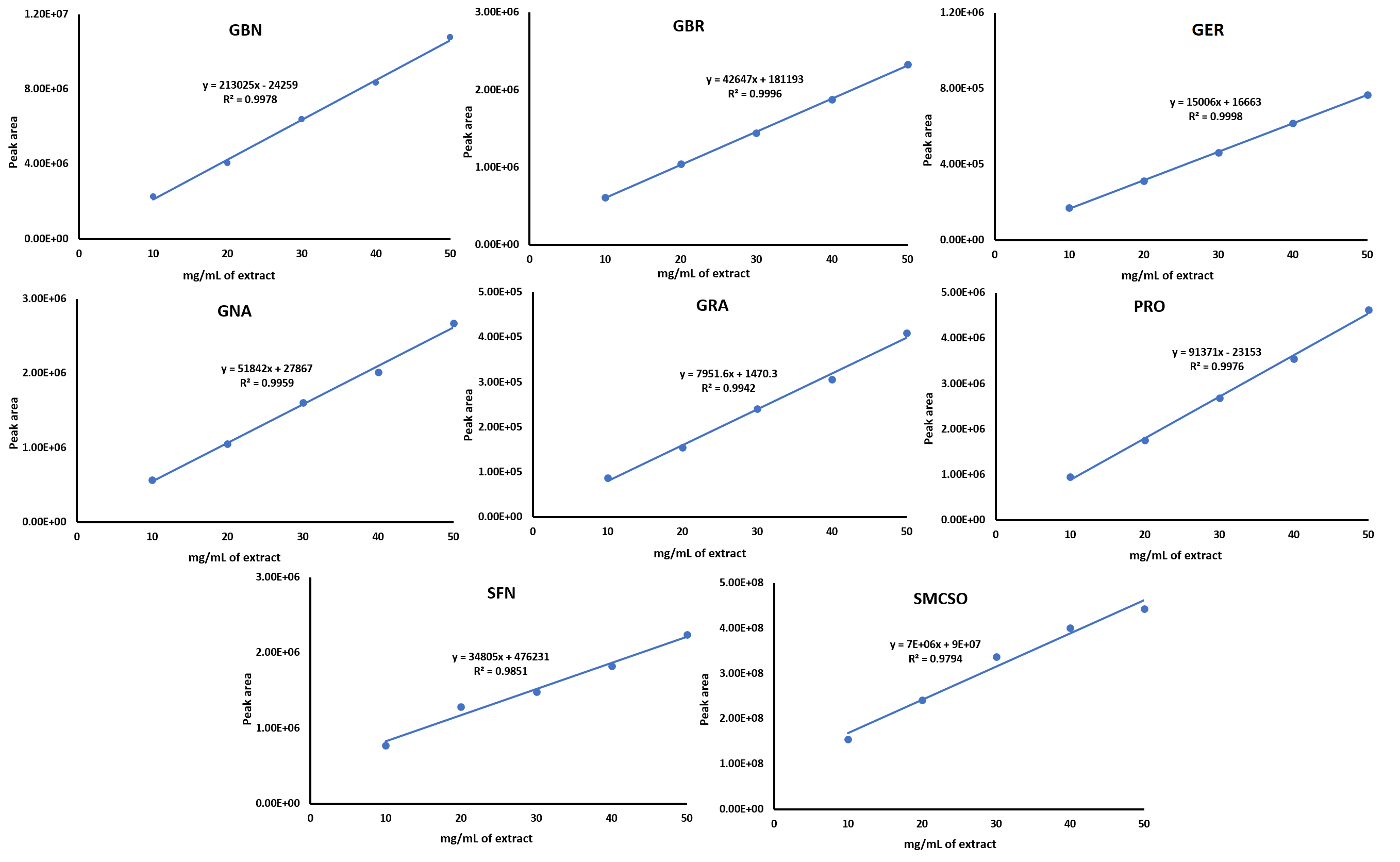


**Fig. S3.** Test the linearity of target analytes at different extraction concentrations in Chinese cabbage.

**
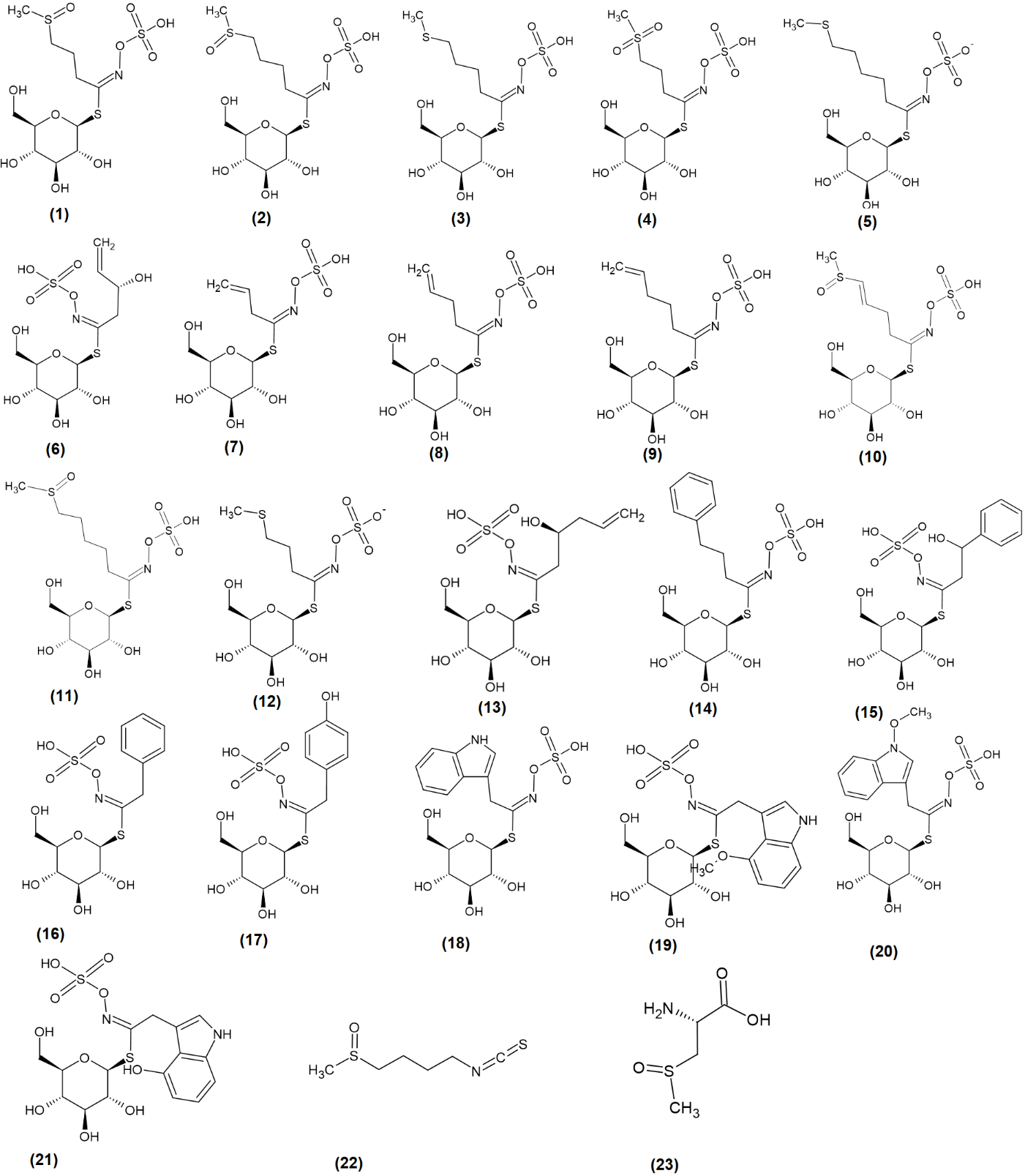
**

**Fig. S4.** Structures of (1) glucoiberin; (2) glucoraphanin; (3) glucoerucin; (4) glucocheirolin; (5) glucoberteroin; (6) progoitrin; (7) sinigrin; (8) gluconapin; (9) glucobrassicanapin; (10) glucoraphenin; (11) glucoalyssin; (12) glucoiberverin; (13) gluconapoleiferin; (14) gluconasturtiin; (15) glucobarbarin; (16) glucotropaeolin; (17) sinalbin; (18) glucobrassicin; (19) 4-methoxyglucobrassicin; (20) neoglucobrassicin; (21) 4-hydroxyglucobrassicin; (22) sulforaphane; (23) S-methyl-L-cysteine sulfoxide.

**References**

Al-Gendy, A. A., Nematallah, K. A., Zaghloul, S. S., & Ayoub, N. A. (2016). Glucosinolates profile, volatile constituents, antimicrobial, and cytotoxic activities of Lobularia libyca. *Pharmaceutical biology, 54*(12), 3257-3263. <https://doi.org/10.1080/13880209.2016.1223146>.

Fabre, N., Poinsot, V., Debrauwer, L., Vigor, C., Tulliez, J., Fourasté, I., & Moulis, C. (2007). Characterisation of glucosinolates using electrospray ion trap and electrospray quadrupole time-of-flight mass spectrometry. *Phytochemical Analysis, 18*(4), 306-319. <https://doi.org/10.1002/pca.983>.

Glauser, G., Schweizer, F., Turlings, T. C. J., & Reymond, P. (2012). Rapid Profiling of Intact Glucosinolates in Arabidopsis Leaves by UHPLC-QTOFMS Using a Charged Surface Hybrid Column. *Phytochemical Analysis, 23*(5), 520-528. <https://doi.org/10.1002/pca.2350>.

Hwang, I. M., Park, B., Dang, Y. M., Kim, S.-Y., & Seo, H. Y. (2019). Simultaneous direct determination of 15 glucosinolates in eight Brassica species by UHPLC-Q-Orbitrap-MS. *Food Chemistry, 282*, 127-133. <https://doi.org/https://doi.org/10.1016/j.foodchem.2018.12.036>.

Liang, X., Lee, H. W., Li, Z., Lu, Y., Zou, L., & Ong, C. N. (2018). Simultaneous Quantification of 22 Glucosinolates in 12 Brassicaceae Vegetables by Hydrophilic Interaction Chromatography–Tandem Mass Spectrometry. *ACS Omega, 3*(11), 15546-15553. <https://doi.org/10.1021/acsomega.8b01668>.

Maldini, M., Baima, S., Morelli, G., Scaccini, C., & Natella, F. (2012). A liquid chromatography-mass spectrometry approach to study “glucosinoloma” in broccoli sprouts. *Journal of Mass Spectrometry, 47*(9), 1198-1206. <https://doi.org/10.1002/jms.3028>.

Millán, S., Sampedro, M. C., Gallejones, P., Castellón, A., Ibargoitia, M. L., Goicolea, M. A., & Barrio, R. J. (2009). Identification and quantification of glucosinolates in rapeseed using liquid chromatography–ion trap mass spectrometry. *Analytical and Bioanalytical Chemistry, 394*(6), 1661-1669. <https://doi.org/10.1007/s00216-009-2823-8>.

Rochfort, S. J., Trenerry, V. C., Imsic, M., Panozzo, J., & Jones, R. (2008). Class targeted metabolomics: ESI ion trap screening methods for glucosinolates based on MSn fragmentation. *Phytochemistry, 69*(8), 1671-1679. <https://doi.org/https://doi.org/10.1016/j.phytochem.2008.02.010>.

Wu, W., Chen, J., Yu, D., Chen, S., Ye, X., & Zhang, Z. (2021). Analysis of Processing Effects on Glucosinolate Profiles in Red Cabbage by LC-MS/MS in Multiple Reaction Monitoring Mode. *Molecules, 26*(17), 5171. <https://www.mdpi.com/1420-3049/26/17/5171>.

Yu, X., Ma, F., Zhang, L., & Li, P. (2020). Extraction and Quantification of Sulforaphane and Indole-3-Carbinol from Rapeseed Tissues Using QuEChERS Coupled with UHPLC-MS/MS. *Molecules, 25*(9). <https://doi.org/10.3390/molecules25092149>.
